# Composites Containing Nanohydroxyapatites and a Stable TEMPO Radical: Preparation and Characterization Using Spectrophotometry, EPR and ^1^H MAS NMR

**DOI:** 10.3390/ma15062043

**Published:** 2022-03-10

**Authors:** Natalia Byra, Sylwester Krukowski, Jaroslaw Sadlo, Waclaw Kolodziejski

**Affiliations:** 1 Department of Analytical Chemistry, Medical University of Warsaw, Banacha 1, 02-097 Warsaw, Poland; sylwester.krukowski@wum.edu.pl (S.K.); waclaw.kolodziejski@wum.edu.pl (W.K.); 2 Institute of Nuclear Chemistry and Technology, Dorodna 16, 03-195 Warsaw, Poland; j.sadlo@ichtj.waw.pl

**Keywords:** hydroxyapatite, adsorption, nitroxide radical, physicochemical properties, biomaterial

## Abstract

Hydroxyapatite is the main constituent of mammalian hard tissues. Basic applications of synthetic hydroxyapatites include bone and dental implantology and drug delivery systems. The study of hydroxyapatite surface properties could give greater insight into the processes of bone mineralization and degradation. Nitroxide radicals are stable radicals that exhibit anticancer and antioxidative properties and are often used as spin probes to study the dynamics of complex biological systems. In this work, we attempted to adsorb the stable 2,2,6,6-tetramethylpiperidine-1-oxyl radical (TEMPO) on two hydroxyapatites (HAs) differing in specific surface area and the degree of hydration. The adsorption was carried out from cyclohexane, 1-chlorobutane and water. The solutions after adsorption were studied spectrophotometrically, while the obtained composites were characterized via NMR and EPR spectroscopy. The results show that it is possible to reproducibly obtain fairly stable composites, where the main factors influencing the adsorbed amount of the radical are solvent polarity and specific surface area of hydroxyapatite. The Langmuir isotherm was determined to be the most suitable adsorption model. The analysis of EPR and NMR spectra allowed us to determine the distribution of the TEMPO molecules on the hydroxyapatite surface, as well as a probable adsorption mechanism. The HA/TEMPO composites could potentially be used to study certain properties of hydroxyapatite surfaces with EPR spectroscopy. They could also be used as fillers after hard tissue surgery, as well as metal-free MRI contrasts.

## 1. Introduction

Due to the high occurrence rate of bone and teeth diseases, bone substitute biomaterials are extensively researched nowadays. Carbonated calcium-deficient hydroxyapatite, which belongs to the group of phosphate minerals called apatites, is the main constituent of mammalian hard tissues. Therefore, synthetic apatites can be used to obtain dental and bone implants as well as to fill bone defects. Other applications include the production of controlled drug delivery systems and non-viral gene therapy vectors, as well as the removal of heavy metals from the environment (especially water) and catalysis [1,2,3,4,5,6].

Biological apatites have a high specific surface area (SSA), are nanocrystalline and non-stoichiometric—some of the calcium ions are replaced by sodium, magnesium, potassium or strontium cations. The locations of phosphate ions and hydroxyl ions can be occupied by carbonate ions named B and A, respectively. Additionally, the amount of hydroxyl groups diminishes. However, the reference compound in this group is stoichiometric hydroxyapatite, HA (Ca_10_(PO_4_)_6_(OH)_2_). It is a suitable material for preliminary research on hydroxyapatites [7].

The biological responses of hydroxyapatites and their effectiveness as drug delivery systems are highly dependent on their physicochemical properties, such as their crystal shape, size, morphology and functional groups [8]. The key to understanding and predicting physicochemical and biological properties of nanocrystalline hydroxyapatites lies in the composition of their hydrated surface layer. This is an interface between the crystalline apatite core and its external environment, which is typical of hydroxyapatites with nano-sized crystals. This layer contains water particles and labile, easily exchangeable ions. Due to charge asymmetry, the layer determines the adsorption capacity of hydroxyapatite and plays an active role in maintaining calcium and phosphorus homeostasis [9]. Knowing the exact qualitative and quantitative composition of the hydrated layer and its changes could broaden the knowledge of pathomechanisms underlying hard tissue diseases. No efficient method enabling this has been developed so far.

Nitroxide radicals, such as TEMPO (2,2,6,6-tetramethylpiperidine-1-oxyl), exhibit unique properties that distinguish them from the majority of free radicals. The first aspect is their relatively high stability, which they owe predominantly to the presence of alkyl groups attached to alpha carbons. These groups form steric hindrance around the >N-O· paramagnetic center carrying an unpaired electron, which prevents the otherwise fast radical recombination, as well as its reactions with other chemical compounds. The stability of nitroxide radicals is also enhanced due to spin density delocalization between the two atomic centers, which is reflected by two resonance structures: >Ṉ–Ō· ‹—› >·Nꚛ–Ō|ө This is why nitroxide radicals are commonly used for spin trapping and as EPR markers in studies concerning the dynamic properties of membranes, micelles and ionic liquids [10].

Another interesting feature of nitroxide radicals is their redox activity: these radicals can undergo both reduction and oxidation. In living cells, however, they have been shown to behave specifically as antioxidants, mainly through imitating the action of superoxide dismutases, a group of metalloenzymes converting harmful superoxide anion radicals (O_2_^−^) into O_2_ and H_2_O_2_. In this regard, the possibility of using these radicals under certain conditions associated with oxidative stress has been studied both in vitro and in vivo. Such pathological states are, among others, inflammations, neurodegenerative diseases, tissue damage due to hypoxia, cataracts, obesity and radiotherapy side effects. It has also been shown that some nitroxide radicals, especially 4-hydroxy-TEMPO, exhibit anticarcinogenic activity in that in cancer cells they act as oxidants and possibly p53 protein activators, which leads to apoptosis. On the other hand, their ability to lower oxidative stress resulting from reactive oxygen species (ROS) in normal cells is used in chemoprevention [11,12,13].

TEMPO, as well as other nitroxide radicals, can participate in hydrogen and coordinate bonds and can be attached to the surfaces of miscellaneous macromolecules. If its paramagnetic character is not lost during the process, using EPR spectroscopy it is possible to study the surfaces of the macromolecules and the dynamics of the radicals on them, since the EPR spectrum shape changes depending on the environment of the radicals and their mobility and interactions [14]. Other applications of nitroxide radicals include functional EPR imaging, catalysis of oxidation reactions, potentially shortening NMR spectra acquisition times and serving as MRI contrast agents [13,15,16].

In this paper, we carried out the adsorption of TEMPO on two commercially available hydroxyapatites of different specific surface areas. The adsorption was performed in three solvents varying in polarity: cyclohexane, 1-chlorobutane and water. This was monitored spectrophotometrically using a UV band of TEMPO. The structure of the hydroxyapatite/TEMPO composites was characterized with magnetic resonance methods, namely electron paramagnetic resonance (EPR) and solid-state proton nuclear magnetic resonance (^1^H MAS NMR), where EPR allowed the observation of the free radical, while ^1^H MAS NMR was used for the analysis of proton-containing diamagnetic species, such as water, hydroxyl groups and residual organic solvents. The aim of this work was to develop a reproducible adsorption method and create a theoretical model of the adsorption process, as well as to determine the distribution and dynamics of the radical molecules in composites. In addition, we wanted to find out whether the TEMPO radical could be used to study the surfaces of hydroxyapatites. There have been numerous reports on combining hydroxyapatites with paramagnetic entities, such as iron oxide nanoparticles and paramagnetic cations [17,18,19]; however, to the best of our knowledge, attaching nitroxide radicals to the surfaces of hydroxyapatites has not been attempted so far.

In the future, we want to tailor the HA/TEMPO composites for biomedical applications (bone regeneration with simultaneous anticancer action).

## 2. Materials and Methods

### 2.1. Materials

The TEMPO radical (2,2,6,6-tetramethylpiperidine-1-oxyl) was purchased from Sigma Aldrich (St. Louis, MO, USA) (purity: 98%) and was used without further purification.

The two commercially available, phase-pure and unsubstituted hydroxyapatites (Ca_10_(PO_4_)_6_(OH)_2_) used in this study had SSA values of 83.3 m^2^·g^−1^ and 259 m^2^·g^−1^. They will be further referred to as HA83 and HA259, respectively. Apart from the information provided by the manufacturers, both hydroxyapatites were additionally characterized using transmission electron microscopy (TEM) and HA83 was subjected to thermogravimetric analysis with differential scanning calorimetry (TGA-DSC).

HA83 was purchased from Calbiochem. Its average crystal size, obtained from TEM 2D measurements, was 117.7 ± 5.6 nm × 52.3 ± 2.9 nm. According to the TGA-DSC analysis (Figure S1), its total water content and the amount of surface water were 3.63 and 2.50 wt%, respectively (the surface water can be eliminated during heating up to 200 °C [20]).

Anhydrous HA259 was obtained from the Nanostructure Laboratory of the Institute of High-Pressure Physics (Polish Academy of Sciences). Its original commercial name was GoHAP1. The average crystal size, calculated from TEM planar images, was 19.1 ± 0.8 nm × 8.5 ± 0.3 nm. To minimize the contact of the material with air, HA259 was stored in an efficient desiccator (glass desiccator with anhydrous CaCl_2_ and CoCl_2_ as an indicator) and all the operations on it were performed in a glove bag (purchased from Sigma Aldrich) under argon. Therefore, under the applied conditions, HA259 used in this study is considered anhydrous.

In our work, the following solvents were used: cyclohexane, purchased from Sigma Aldrich (purity ≥99%); 1-chlorobutane, purchased from Avantor Performance Materials (purity: for HPLC) and from Acros Organics (purity: for HPLC); doubly distilled water. Hereafter, the solvents are given the symbols CH, BuCl and W, respectively. CH and BuCl were used without further purification. The water used complied with ISO 3696 (grade 1) standards.

### 2.2. Adsorption Studies

By diluting a TEMPO stock solution (16 mM), we prepared a series of solutions in a given solvent (CH, BuCl or W) with increasing radical concentrations (c_0_), ranging from 0.5 mM to 16.0 mM. The absorbance of the obtained solutions was measured at a chosen analytical wavelength in order to plot a calibration curve (A = f(c_0_)). The reference cell contained pure solvent.

Here, 10.0 mL of each solution was added to 100 mg of a given hydroxyapatite. The obtained suspensions were shaken for 3 h at 298 K in closed Teflon test tubes at 250 rpm and then centrifuged (3500 rpm, 10 min). The absorbance of each supernatant was measured at the analytical wavelength chosen previously for the calibration curve. During this procedure, the reference cell contained pure solvent shaken earlier with the hydroxyapatite (under the same conditions as other solutions). The equilibrium concentration of TEMPO in the solutions after adsorption (c_e_) was calculated from the calibration curve.

The precipitates containing HA83 were left to dry overnight at room temperature (RT) under a fume hood and were later subjected to EPR and ^1^H NMR studies. The precipitates containing HA259, in turn, were left to dry for two days in a desiccator flushed with argon. They were also subjected to further studies. In the rest of the work, the obtained composites are referred to in the following way: HA83/TEMPO(X) and HA259/TEMPO(X), where X denotes CH, BuCl or W, depending on the solvent used for adsorption.

Experimental adsorption isotherms were plotted as a relationship between the TEMPO concentration after adsorption (c_e_, equilibrium concentration) and the amount of TEMPO adsorbed on hydroxyapatite per unit mass (q_e_), calculated from the following equation:

q_e_ = (c_o_ − c_e_)[mmol·L^−1^] × V[L]/W_HA_[g] (1)

where V is the volume of the TEMPO solution added (0.01 L) and W_HA_ is the mass of HA used for adsorption (0.1 g).

The procedures described above, including the preparation of a calibration curve, were repeated 3 times for each solvent. To plot the adsorption isotherms, average values of c_e_ and q_e_ were taken.

### 2.3. Spectrophotometric Measurements

The spectrophotometric studies were carried out at 298 K using a Shimadzu UV 1800 spectrophotometer. The quartz cuvettes used had an optical path length of 0.05 cm. In this work, we focused on the electronic transition of TEMPO in the UV region (λ_max_ = 240.5–243.5 nm; the peak wavelength was slightly dependent on the solvent). The transition in the visible (VIS) region was useless in this case because of the low molecular absorption coefficient, such that for the applied low radical concentrations, the visible band had insufficient absorbance for the analytical work (below 0.1). The spectra of TEMPO in the studied solvents are presented in Figure S2.

The choice of solvents was dictated by their polarity and absorbance cutoff wavelength. For CH, BuCl and W, the UV cutoff values were 200, 220 and 190 nm, respectively, which were below the chosen analytical wavelength. The polarity increased in the given order.

### 2.4. EPR Measurements

The continuous-wave EPR measurements were performed for 0.5 mM TEMPO solutions (in CH, BuCl and W) and for dried HA/TEMPO composites obtained after adsorption. They were carried out at 294 K using an X-band Bruker EMXPlus spectrometer.

The measurement parameters were as follows: microwave power 1 mW, modulation amplitude 1 G, conversion time 10 ms, time constant 1.28 ms, sweep width 300 G, sweep time 30 s, number of points 3000. Every sample was weighted before the measurement.

The intensity of EPR signals was determined by double integration of the spectrum with the use of the KaleidaGraph program, version 3.5, 2000 (Synergy Software; Reading, PA, USA). All three repetitions were analyzed and the average integral intensities were presented. The fitting of EPR spectra was performed with the MultiComponent program developed by Altenbach [21], with the use of the Levenberg–Marquardt algorithm hybrid method. For this purpose, the number of spectral points was reduced to 1500.

### 2.5. NMR Measurements

The solid-state ^1^H NMR spectra at 298 K and the resonance frequency of 400 MHz were recorded using a Bruker Avance 400 WB spectrometer and a simple pulse-acquired (Bloch decay) pulse sequence. The measurements were carried out with magic angle spinning (MAS) at 7.5 kHz, using 4 mm ZrO_2_ rotors driven by dry air. The spectra were acquired with a recycle delay of 10 s, a π/2 pulse of 2.6 μs and 8 scans. The spectral background resulting from the rotor and the MAS probe was carefully subtracted.

### 2.6. Modeling of Adsorption Isotherm Data

To analyze the collected data and fit model isotherms to the obtained experimental adsorption points, we used MS Excel for Microsoft 365 MSO and KaleidaGraph version 3.5, 2000.

### 2.7. Thermogravimetric Analysis with Differential Scanning Calorimetry (TGA-DSC)

The TGA-DSC analysis was carried out in argon up to 1000 °C using a SDT Q600 V20.9 Build 20 thermogravimetric analyzer.

### 2.8. Transmission Electron Microscopy (TEM)

The 2D TEM measurements were carried out using a JEM 1400 JEOL transmission microscope. A drop of a sample suspension in ethanol was placed on a Cu grid covered with a formvar film, allowed to dry and analyzed under the accelerating voltage of 80 kV. The average crystal size values were calculated from the data for 105 crystals randomly chosen in the fields of representative view. The exemplary TEM images for both HAs are available in the Supplementary Materials (Figure S3).

## 3. Results

### 3.1. Adsorption Isotherms

#### 3.1.1. Experimental Adsorption Isotherms

**Figure 1:**
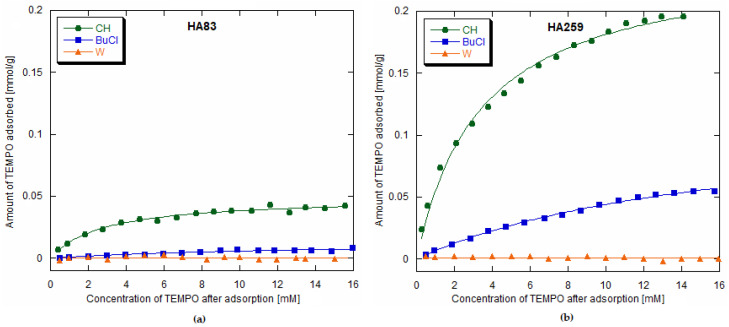
Adsorption isotherms of 2,2,6,6-tetramethylpiperidine-1-oxyl radical (TEMPO) on hydroxyapatites (HAs) from different solvents (Langmuir plots): (**a**) on HA83; (**b**) on HA259.

The obtained averaged experimental points with fitted model isotherms are depicted in Figure 1. The best fit was achieved for the Langmuir model of adsorption. Indeed, according to previous studies concerning the adsorption of organic molecules on hydroxyapatites, this model was among the most frequently chosen ones, along with the Freundlich model [22].

Since both the Langmuir and Freundlich models gave fairly decent fits, we compared them in terms of accuracy. It was proven that determination coefficient (R^2^) is not an appropriate indicator for nonlinear models [23]. Therefore, apart from the R^2^ values, we based our reasoning on the chi-square statistics (χ^2^), which is seemingly useful for two-parameter adsorption isotherms [24], and on the small-sample corrected Akaike information criterion (AIC_c_), considered to be one of the best measures of fit accuracy for adsorption isotherms, irrespective of the number of model parameters [25]. The AIC_c_ values for the models in question and the evidence ratio values directly connected with them were calculated and interpreted in accordance with the work by Motulsky and Christopoulos [26] (pp. 143–148). The obtained parameters are presented in Table 1. In each case, the Langmuir model gave a better fit.

The Langmuir isotherm is described by the following equation:

(2) $$\frac{q_{e}\;=\;Q_{0}\;K_{L}\times {\;C}_{e}}{1+K_{L}\times {\;C}_{e}}$$

where K_L_ [L·mmol^−1^] is the Langmuir constant, denoting affinity to the adsorbent; Q_0_ [mmol·g^−1^] is the maximum monolayer adsorption capacity; C_e_ [mM] is the equilibrium concentration of TEMPO after adsorption; q_e_ [mmol·g^−1^] is the amount of TEMPO adsorbed per HA unit mass at a given point [27]. The obtained Langmuir isotherm parameters for HA83 and HA259 and the three studied solvents are juxtaposed in Table 2.

The table also contains the calculated molar adsorption coefficients of TEMPO in the studied solvents. As expected, more TEMPO molecules adsorbed onto the HA of higher specific surface area.

For both hydroxyapatites, the amount of TEMPO adsorbed diminished with the increase in solvent polarity. Additionally, the obtained K_L_ values indicated that the affinity towards a given HA also decreased with the increase in solvent polarity. In the case of the adsorption from water, the amounts of the radical adsorbed on HA were very slight. Therefore, it was not possible to determine accurate q_e_ values or to fit a model adsorption isotherm for this solvent.

#### 3.1.2. Coverage of Hydroxyapatite Surface by TEMPO Molecules

To investigate how much space the TEMPO molecules had on the surfaces of hydroxyapatites, we calculated the HA surface area per one adsorbed radical molecule. The calculations were based on the SSA and q_e_ values under the assumption that TEMPO was distributed evenly on the apatite surface and that this surface was entirely available for adsorption, although for HA83 competition with water molecules was expected. For CH and BuCl, the relationships between the initial radical concentration and the HA surface area per one TEMPO molecule resembled the reversed adsorption isotherms for both HAs (Figure S4). In the case of water, there was no regular dependence pattern and the points were distributed chaotically due to the very low adsorption intensity from this solvent. In Table 3, we present numerical values for the HA surface area per one TEMPO molecule for three chosen initial radical concentrations (experimental points), as well as for the calculated maximum adsorption capacities (theoretical values) for the used hydroxyapatites and the organic solvents. The TEMPO molecule dimensions, obtained from diffusion kinetics, were 0.65 × 0.81 × 0.89 nm [28]. For the purposes of this work, considering the van der Waals volume of 0.202 nm^3^ [29], it was assumed that the TEMPO molecule is a sphere with a radius of 0.364 nm and its cross-section area amounts to approximately 0.42 nm^2^. Since all values in Table 3 are at least 4 times larger than the radical cross-section area, it is reasonable to assume that there was enough HA surface available for TEMPO to form a monolayer.

It was also of great interest how the presence of water on HA83 interfered with the TEMPO adsorption. Based on the TGA-DSC results (2.50 wt% of surface water) and assuming that one water molecule adsorbed on hydroxyapatite effectively covers an area of 0.115 nm^2^ [30], we calculated that there were about 10 water molecules per 1 nm^2^ surface area of HA83, meaning that all of the HA83 surface was covered by a monolayer of chemisorbed water [30].Therefore, in order to attach itself to this surface, the radical had to displace some water from it. Thus, the competition of TEMPO with water for the apatite surface should be further considered for HA83.

### 3.2. EPR Study

#### 3.2.1. General Analysis of the EPR Spectra

**Figure 2:**
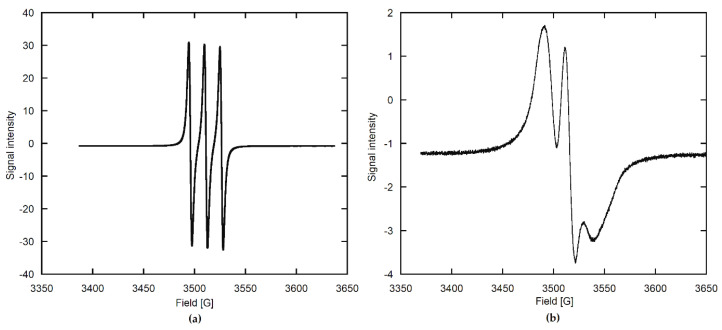
The EPR spectra of TEMPO in a very dilute solution and after adsorption on an anhydrous HA surface: (**a**) TEMPO in the 0.5 mM cyclohexane (CH) solution, unrestricted motion; (**b**) TEMPO adsorbed on HA83 from the 0.2 mM CH solution, with the apatite previously dehydrated at 200 °C, restricted motion.

**Figure 3:**
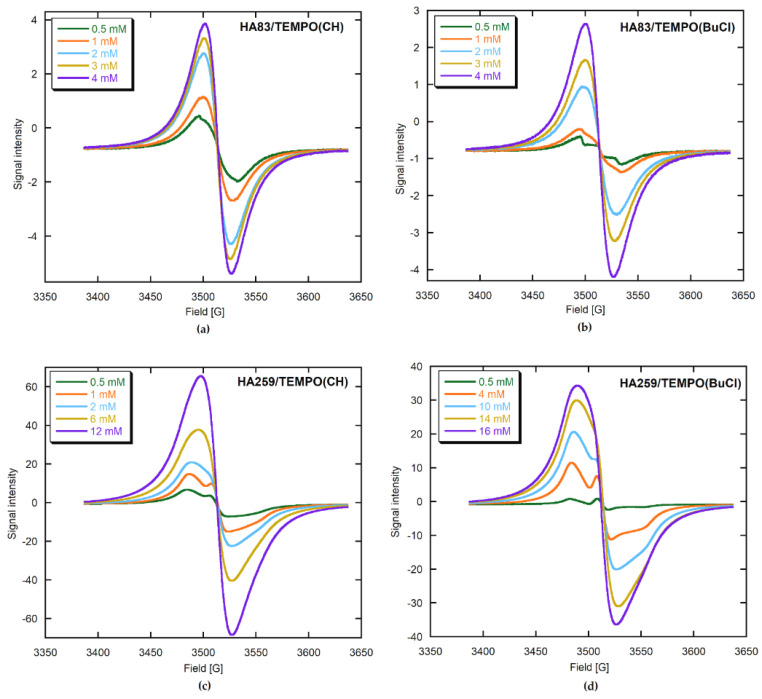
The EPR spectra of TEMPO from the obtained composites showing signal dependence on the initial radical concentration in the organic solvent: (**a**) after adsorption on HA83 from CH; (**b**) after adsorption on HA83 from 1-chlorobutane (BuCl); (**c**) after adsorption on HA259 from CH; (**d**) after adsorption on HA259 from BuCl.

**Figure 4:**
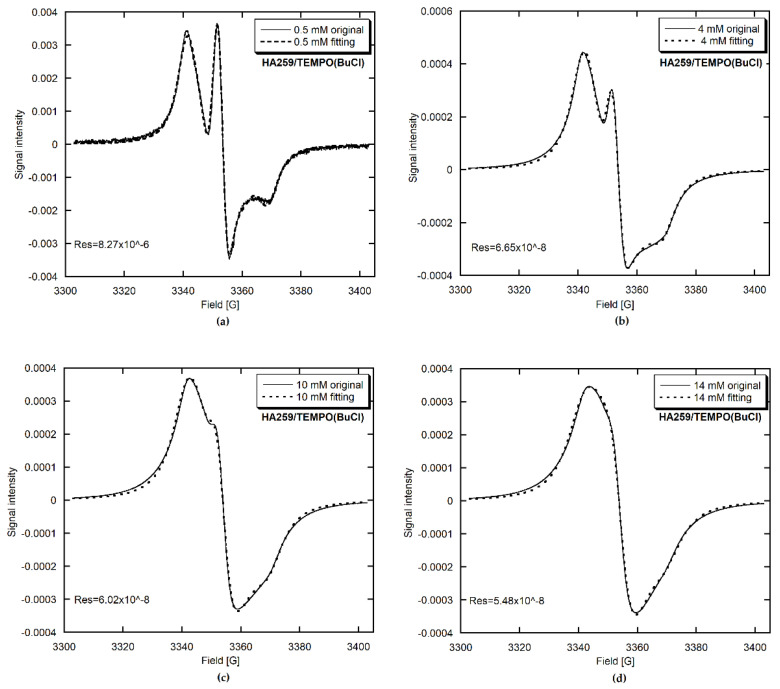
The original and simulated EPR spectra for the HA259/TEMPO(BuCl) composites prepared using four different initial TEMPO concentrations in BuCl: (**a**) 0.5 mM; (**b**) 4 mM; (**c**) 10 mM; (**d**) 14 mM.

**Figure 5:**
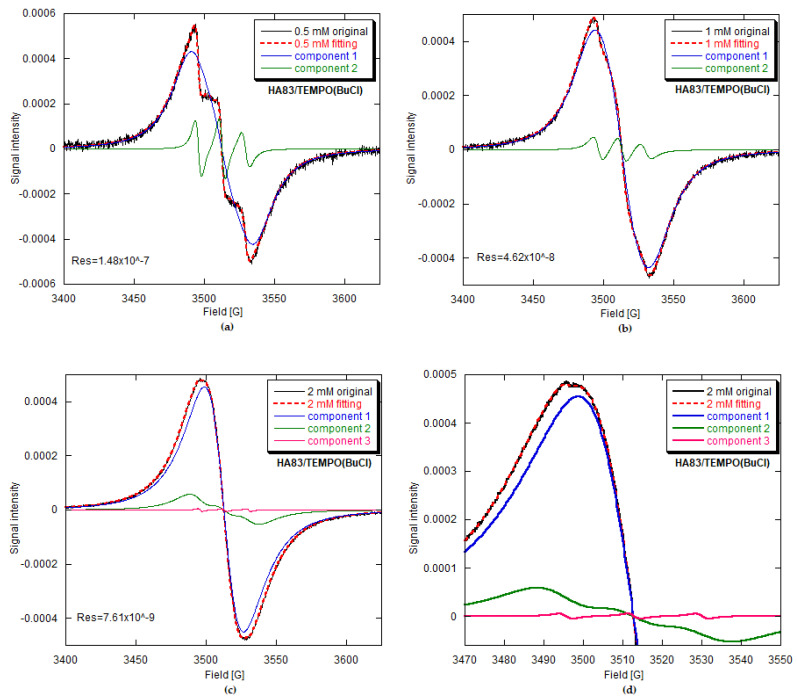
The original and simulated EPR spectra of the HA83/TEMPO(BuCl) composites prepared using three different initial TEMPO concentrations in BuCl: (**a**) 0.5 mM; (**b**) 1 mM; (**c**) 2 mM; (**d**) a magnified region of the 2 mM spectrum, justifying a contribution from the third spectral component.

**Figure 6:**
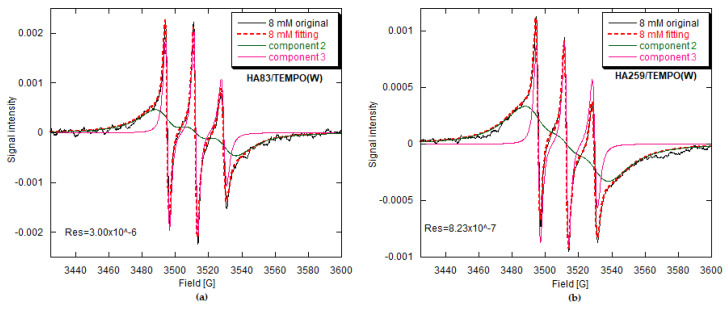
The original and simulated EPR spectra of the composites prepared using the 8 mM aqueous solutions of TEMPO: (**a**) HA83/TEMPO(W); (**b**) HA259/TEMPO(W).

The TEMPO radical retained its paramagnetic character after adsorption. The pertinent EPR spectra of the HA/TEMPO composites were easy to acquire and of sufficient quality to be analyzed in terms of the material structure, adsorption mechanisms and molecular dynamics of TEMPO on the HA surface. We had only a few doubts concerning the comparisons of signal intensities, which are discussed later (Section 3.2.5 and Section 4.5). The composites prepared via the TEMPO adsorption from CH and BuCl were fairly stable in time if they were stored with no contact with light, and in the case of the HA259/TEMPO materials, in an efficient desiccator under argon. The HA/TEMPO(W) materials were less stable but also less interesting because of the very small radical concentration. Only in a few cases was it observed that the radical could change its adsorption environment with time. Representative EPR spectra measured in our work are presented in Figure 2, Figure 3, Figure 4, Figure 5 and Figure 6. The term “initial radical concentration” used in the figures of the EPR section corresponds to c_0_ in Section 2.2. and designates the initial concentration of TEMPO in the solutions, from which its adsorption was carried out. Hereafter, the peak-to-peak linewidth ∆H_pp_ is defined as the distance between positive and negative peaks of the derivative EPR line.

The EPR spectra of stable nitroxide radicals have already been successfully used in materials science [31]. Two selected spectra from our work, measured in extreme motion cases, are presented in Figure 2.

The spectrum of TEMPO in a very dilute solution (Figure 2a), where the motion of the radical is unrestricted, consists of three equal, sharp lines, resulting from the coupling between the unpaired electron and the ^14^N nucleus of spin 1 (hyperfine structure, HFS). In this case, the radical motion is almost isotropic, so the tensorial physical quantities, such as tensors **g** (signal position) and **A** (signal hyperfine splitting), are averaged to isotropic values equal to one-third of the respective tensor traces.

The spectrum of TEMPO adsorbed on anhydrous apatite (Figure 2b) indicates considerably slower dynamics of the radical molecules and rather small lateral interactions between adjacent species (spectral features are clearly resolved). The radical molecule is fixed to the adsorbent surface and experiences substantially restricted motion, here being quite close to the rigid case with the TEMPO motion being completely frozen. In such circumstances, full **g** and **A** tensors should be considered.

Between those extreme cases, which differ in the TEMPO molecular motion, numerous intermediate conditions can be found in our study (Figure 3, Figure 4, Figure 5 and Figure 6). Therefore, the resulting EPR spectra are best analyzed using appropriate computer programs. Additional variables to be accounted for are the magnetic dipole–dipole (DD) interaction between unpaired electrons and the Heisenberg spin exchange (SE) [32,33,34,35,36,37,38]. The former operates through space and the latter requires the molecular orbitals of unpaired electrons to be overlapped (direct spin exchange, negligible for the interspin distances over 1.5 nm) [39]. Both phenomena are dependent on the radical dynamics and distribution in a sample.

Unfortunately, line broadening blurs the EPR hyperfine structure. The DD and SE mechanisms operate simultaneously, both affecting the linewidth and both being more pronounced if radical molecules move closer to each other. The DD interaction causes only line broadening, while the SE interaction can either enlarge or reduce the linewidth. The exchange broadening appears with the onset of the SE interaction (weak exchange). This increases with the increase in the radical concentration, along with the SE rate, gradually obscuring the hyperfine structure. At some stage the hyperfine structure becomes completely unresolved; that is, it collapses to a single, broad, featureless line, reaching a maximum width. Thereafter, the exchange narrowing is observed (strong exchange).

The exchange narrowing reduces the linewidth to 11.5 G, which is the value obtained for pure crystalline TEMPO [40]. In our spectra the linewidth after the hyperfine structure collapse never dropped below 21 G and a spectral component with a linewidth of 11.5 G was never isolated. Evidently, the crystals of pure TEMPO are absent from the HA/TEMPO composites, especially as TEMPO undergoes sublimation even at RT and cannot simply coexist with HA as a separate crystalline phase.

For the lowest radical concentrations, the EPR spectra of HA259/TEMPO(CH) and HA259/TEMPO(BuCl) (Figure 3) resemble those of the restricted motion case (Figure 2b). On the other hand, the spectra of HA/TEMPO(W) for both apatites include sharp triplets (Figure 6), demonstrating rather high mobility of TEMPO, which is unexpected in the solid state. No more can be said about TEMPO mobility in the HA/TEMPO composites without a rigorous computer analysis of their spectra. However, it is obvious from the visual inspection that the EPR spectra of our composites are dependent on HA, the solvent, the presence of water and the concentration of TEMPO (Figure 3, Figure 4, Figure 5 and Figure 6). The spectra can be directly analyzed in view of the resolution of the ^14^N hyperfine structure, the TEMPO concentration at which this structure collapses and the behavior of a peak-to-peak linewidth ∆H_pp_ after the collapse, measured directly from the experimental spectrum.

The EPR spectra of the HA259/TEMPO composites prepared by TEMPO adsorption from CH and BuCl are much more intense than the spectra of the corresponding HA83/TEMPO composites (Figure 3), indicating better adsorption performance of TEMPO on HA259 than on HA83. In the case of the TEMPO adsorption from the 8 mM aqueous solutions, the situation seems to be reversed (Figure 6), but all the spectra of HA/TEMPO(W) were extremely weak and prone to quantitative errors. Regarding the solvent-dependent trends in the spectral intensities, they obeyed the following order for both HA259/TEMPO and HA83/TEMPO (Figure 3 and Figure 6; Table S1): CH > BuCl >> W. This series indicates a decrease in the TEMPO loading with an increase in solvent polarity, which is consistent with the UV–Vis adsorption studies and the fitted isotherms.

In the HA/TEMPO(W) cases, there are evidently two spectral components (Figure 6), so the presence of water makes the spectra more complex. Such multicomponent spectra are also obtained for HA83/TEMPO(CH) and -(BuCl), but their components can only be revealed by a computer program (see later).

In Figure 3, it is also visible that for HA259/TEMPO(CH), the HFS is hardly resolved for the 0.5 and 1 mM TEMPO in CH. At about 2 mM the HFS collapses and then the resulting structureless line narrows: ∆H_pp_ decreases from 38.4 to 27.4 G in the 2–16 mM range. For HA259/TEMPO(BuCl), the HFS is well resolved even up to the initial concentration of 4 mM. The HFS gradually loses resolution in the 0.5–12 mM concentration range and collapses at about 13 mM. At first, the resulting structureless line broadens slightly: ∆H_pp_ increases from 39.4 G at 13 mM to 40.0 G at 14 mM. After this, it narrows from 39.6 G at 15 mM to 36.4 G at 16 mM. For HA83/TEMPO(CH), the HFS is virtually indiscernible at the lowest TEMPO concentration and fully collapses for further data points. The observed broad line gradually narrows: ∆H_pp_ decreases from 28.8 to 23.2 G in the 1–16 mM range. For HA83/TEMPO(BuCl), the HFS is still visible after adsorption from the 0.5 mM TEMPO solution. For the next concentration it is almost indiscernible, while at 2 mM the complete HFS collapse takes place. Thereafter, the observed broad line gradually narrows: ∆H_pp_ decreases from 32.8 to 23.8 G in the 2–16 mM range. Generally, the observed changes in the spectrum shape qualitatively indicate that the Heisenberg spin exchange begins to operate earlier for HA83 than for HA259 and for CH than for BuCl. These findings implicate shorter intermolecular distances between adsorbed TEMPO molecules for HA83 than for HA259 and for CH in comparison to BuCl.

#### 3.2.2. Computer Analysis of the EPR Spectra and Signal Assignments

To characterize the above observations quantitatively and to study potential changes in radical mobility, we performed EPR spectra fittings with Altenbach’s MultiComponent program based on the stochastic Liouville equation and using a modified Levenberg–Marquardt minimization algorithm [21]. Concerning the fitting methodology for slow-motion spectra, we referred to the article of Budil et al. [33]. The chosen fits are presented in Figure 4, Figure 5 and Figure 6. In all cases, the fit accuracy, expressed through the sum of square residuals (Res), was satisfactory. The fitted parameters, either TEMPO-loading-dependent or not, as well as the assumptions made to simplify computations, are given in Table 4.

The TEMPO-loading-dependent parameters included: (1) the Lorentzian linewidth tensor (**W**), corresponding to inhomogeneous line broadening; (2) the Heisenberg spin exchange rate (ν_ex_) governed by the oss parameter; (3) the rotational diffusion tensor (**R**, Brownian diffusion), from which the rotational correlation time was calculated (isotropic τ_c_ and τ_z_ for the axial case). The parameters independent of the free radical amount, fitted only once and kept constant through the rest of the fitting process for a given material, were the following: (4) the **g** factor; (5) the electron–nuclear hyperfine interaction tensor (**A**); (6) the diffusion tilt angle (β_D_), which specifies how much the magnetic axes should be rotated about the rotational diffusion Y axis for the magnetic and rotational diffusion frames to match. According to a widely accepted convention, the magnetic X axis is aligned with the N−O· axis of TEMPO, the Y axis is perpendicular to the X axis and situated in the plane containing the piperidine ring of the >NO· group and the Z axis is located along the 2p_z_ orbital of the nitrogen atom, perpendicular to the aforementioned plane [41].

For the sake of clarity and better understanding, it is assumed that for β_D_ = 90° the z axis of the tensor **R** is colinear with the N−O· axis. If the latter axis was perpendicular to the apatite surface with the nitroxide group attached by means of its oxygen atom and if the TEMPO molecule strongly favored rotational diffusion about this axis (axial **R**), the R_zz_ tensor component would be considerably bigger than the R_xx_ and R_yy_ components, while the corresponding rotational correlation time τ_z_ would be much shorter than τ_x_ and τ_y_. Based on the above reasoning, it can be accepted that if the adsorption of TEMPO took place on a calcium cation and if β_D_ was 90°, the Ca^2+^ ion would interact with two lone electron pairs of the TEMPO oxygen atom (a bifurcated configuration) and the TEMPO mobility would be fully expressed by τ_z_. On the other hand, for β_D_ equal to 45°, the Ca^2+^ ion would be linked to only one of those pairs. The mobility would be still expressed by τ_z_ but the rotating piperidine ring would cover a much larger space around the R_zz_ axis than in the former case.

In the EPR spectra of our HA/TEMPO composites, we were able to discern three kinds of signals, defined in the computer program as spectral components 1–3 (Table 4), each coming from a different TEMPO environment. Hereafter, we mainly focus on their assignments as well as the related structural aspects and dynamics of the adsorbed TEMPO molecules.

For HA259/TEMPO, only one component, labelled as component 1, was present (Figure 4). For HA83/TEMPO(CH or BuCl) (Figure 5, Table S2), this was accompanied by component 2 (up to 21%) and occasionally by component 3 (below 0.1%). Considering the EPR characteristics of component 1, its shape typical of the almost rigid case [32] and its 100% contribution to the spectra of the HA259/TEMPO materials prepared with anhydrous HA, we tentatively assigned this component to the least mobile TEMPO molecules adsorbed directly on the apatite surface, and more specifically on its calcium cations.

Component 3 was observed for TEMPO in water-rich environments, especially for HA259/TEMPO(W) and HA83/TEMPO(W) prepared via the adsorption of TEMPO from aqueous solutions (Figure 6). In those cases, component 3 had a marked share in the EPR spectra at up to approximately 10% (Table 4). It showed up as a sharp triplet with relative line intensities typical of TEMPO in a viscous solution and was best simulated with EPR parameters similar to those of TEMPO dissolved in water, although the **A** tensor turned out to be anisotropic. A completely isotropic hyperfine coupling for component 3 was assumed only in the case of HA83/TEMPO (CH and BuCl) materials and was represented by A_iso_ = 17.28 G, the same as in pure water at RT [42]. Furthermore, the combination of component 3 and a broader and less resolved triplet of component 2 (Figure 6) was reminiscent of the spectrum presented by Ottaviani et al. [32], which had been attributed to TEMPO in “pockets” of water and to the radical molecules not interacting with the adsorbing surface of zeolite. Therefore, we tentatively assigned component 3 to the most mobile TEMPO molecules confined in semiliquid aqueous environments, such as water occupying voids between apatite particles. It was also assumed that those molecules do not interact with the apatite surface.

In terms of the mobility of TEMPO, component 2 should be placed between components 1 and 3. Considering the visual aspects of component 2 (Figure 5 and Figure 6), this mobility is moderate, which is not fully expressed by the τ values (Table 4). Component 2 certainly shows up for materials in which water is present. When TEMPO is adsorbed from aqueous solutions, component 2 is accompanied by the water-related component 3. Additionally, component 2 was observed in the HA83/TEMPO spectra (both the CH and BuCl cases, minor contribution) because HA83 was hydrated. Moreover, this component was clearly visible and dominated in the spectra of composites prepared via the adsorption of TEMPO from aqueous solutions, irrespective of the used HA. Another important fact is that components 2 and 3 come from TEMPO molecules that experience isotropic motion and do not take part in SE (Table 4). The above arguments incline us to tentatively assign component 2 to TEMPO molecules interacting with water molecules adsorbed on the apatite surface. This interaction probably takes place by means of hydrogen bonds.

#### 3.2.3. EPR Parameters of Adsorbed TEMPO

Component 1 allows one to observe the HFS collapse and easily monitor narrowing of the posterior exchange. Since for the HA259/TEMPO materials only component 1 was present, this issue has already been described in Section 3.2.1. For the HA83/TEMPO composites, component 1 had no HFS from the very beginning, so the collapse already occurred at a lower TEMPO concentration than the starting concentration in our work. The linewidth of featureless component 1 decreased from 33.2 to 21.8 G and from 43.6 to 22.8 G for the CH and BuCl adsorption solvents, respectively. Incidentally, the abovementioned behavior of HFS poses an important question as to why the SE interaction is already operative for the low radical loadings for HA259/TEMPO(CH) and HA83/TEMPO (CH and BuCl). The problem can be explained by assuming a crowding of radical molecules on the apatite surface.

For all the materials, the SE rate ν_ex_ obtained for component 1 increased over the whole concentration range studied (Table 4), with a tendency to reach a plateau (Figure S5). It was generally bigger for HA83 than for HA259 and for CH than for BuCl. For HA83, the τ_z_ results were similar for both solvents, while for HA259 this correlation time was generally longer after adsorption from CH than from BuCl, indicating a lower TEMPO mobility in the CH case. The reported values of τ_z_ for component 1 proved that the TEMPO molecules adsorbed directly on HA259 were indeed in the slow motion regime (depending on the source, the slow-motion condition means a rotational correlation time longer than 2–8 ns [32,34,35]).

Considering the above information on the HFS collapse and the SE rate supplied by component 1, one can infer that TEMPO molecules were more crowded on the surface of HA83 than HA259 and after adsorption from CH than from BuCl. However, that crowding effect is not consistent with the greater mobility of TEMPO adsorbed on HA83 from both solvents, as reported by τ_z_ (Table 4). This problem of the surprisingly high mobility of TEMPO adsorbed on HA83 will be covered again in the Discussion.

Component 1 also provided important information on the geometrical arrangement of TEMPO molecules adsorbed on surface calcium cations of HA. The following considerations are valid for the radical adsorption carried out from both CH and BuCl. For HA259/TEMPO, the β_D_ was determined to be 90° for the lowest TEMPO concentration, and for the subsequent fittings it was fixed at this value up to the highest concentration. However, for higher radical concentrations it was also possible to obtain a good fit with different β_D_ values, ranging from 70° to 40°. For HA83/TEMPO, the β_D_ was approximately 54° for all the performed fittings. It follows that there are two possible ways in which the Ca^2+^ cations interact with TEMPO: through only one LP of the TEMPO oxygen atom or simultaneously through two oxygen LPs (see [43]). The single LP configuration dominates for HA83/TEMPO, while the bifurcated configuration prevails for HA259/TEMPO up to moderate TEMPO concentrations in the adsorption solutions. For HA259 materials with the highest surface coverage by TEMPO, the radical molecules are crowded on the apatite surface, which impels a number of them to adopt the single LP configuration.

Component 2 is present in the EPR spectra of HA83/TEMPO for the adsorption from CH, BuCl and W, and in those of HA259/TEMPO(W). Of the abovementioned composites, from a practical perspective, HA83/TEMPO(CH) and HA83/TEMPO(BuCl) are of considerable interest. Component 2 undergoes the HFS collapse at 1 mM and 2 mM for the TEMPO adsorption solutions in CH and BuCl, respectively. This suggests a higher crowding of TEMPO on HA83 when it is adsorbed from CH. Furthermore, it is interesting that this radical crowding occurs for component 2 even at very low TEMPO loadings.

Components 2 and 3 were less informative than component 1, mainly because their fittings were poor and often divergent, especially when component 2 lost its HFS because of efficient SE. Overall, for components 2 and 3, any analysis of the rotational diffusion may be ambiguous, because there were only limited data available on τ_iso_, and τ_iso_ should not be directly compared with τ_z_.

#### 3.2.4. Distances between Molecules of Adsorbed TEMPO

Strictly speaking, these are distances between unpaired electrons of nitroxide groups. They were calculated on the basis of electron–electron magnetic dipolar interactions from the original EPR spectra of HA259/TEMPO. We applied two approaches: depending on ∆H_pp_ of the central line of the TEMPO triplet (method I), as proposed by Sackmann et al. [36] and applied by Ottaviani et al. [32], and based on the d1/d ratio of appropriate distances from the restricted motion spectra (method II) [44,45].

Generally, the interspin distances for the molecules attached directly to the Ca^2+^ cations are in the 1.0–1.6 nm range, while the diameter of the TEMPO molecule is about 0.73 nm. Thus, the crucial structural information is that the TEMPO molecules adsorbed on the apatite surface are very close to one another, even at low radical loadings. The details of the calculation approaches and their limitations are given in the Supplementary Materials.

#### 3.2.5. Integral Intensities of the EPR Spectra

**Figure 7:**
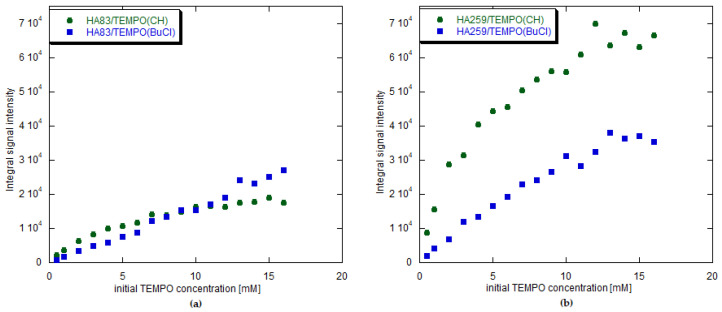
Relationships between the initial TEMPO concentration and the EPR signal integral intensity: (**a**) for HA83/TEMPO composites; (**b**) for HA259/TEMPO composites.

The relationships between the calculated intensities, scaled to the same sample mass, and initial TEMPO concentrations are presented in Figure 7. In the case of water, the amount of TEMPO adsorbed was too small to analyze the spectra even semiquantitatively. In the case of HA259, all findings were consistent with the spectrophotometric measurements. For HA83, however, the results obtained with EPR spectroscopy after adsorption from organic solvents were contradictory to the spectrophotometric findings. The EPR signal intensities showed that the amount of TEMPO adsorbed from BuCl at the initial concentration of 7 mM came close to the amount adsorbed from CH and even exceeded it at higher concentrations. This, in turn, complied with the W parameter values calculated for HA83.

### 3.3. ^1^H NMR Study

#### 3.3.1. HA83

**Figure 8:**
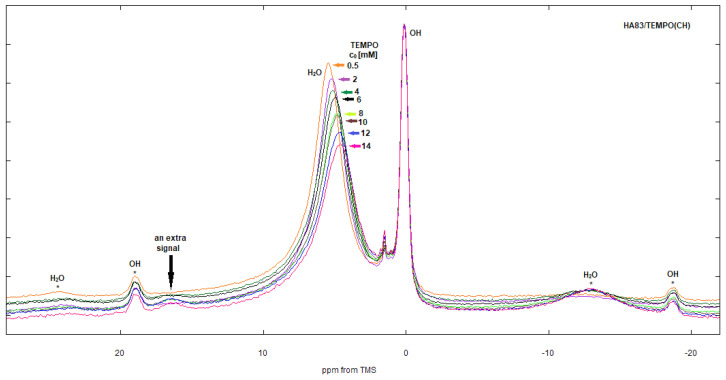
^1^H NMR spectra of the HA83/TEMPO(CH) composites, shown for different TEMPO concentrations c_0_ applied during adsorption. The spectra were normalized to the same sample mass and scaled to the same intensity of the 0 ppm peak. Asterisks denote spinning sidebands.

The proton NMR spectra of the HA83/TEMPO(CH) composites prepared by adsorption from several increasing radical concentrations are depicted in Figure 8. The spectra of the HA83/TEMPO(BuCl) materials showed the same trends, and so are not presented in a separate figure. The HA83/TEMPO(W) samples are not considered here because of insufficient radical loading and the spectral effects being too small.

Each HA83/TEMPO(CH) spectrum contained two main signals (Figure 8): a narrow peak at about 0 ppm from structural hydroxyl groups residing in the apatite interior and a broader peak at around 5.5 ppm from water on the apatite surface. The 5.5 ppm peak evidently broadened and moved towards lower chemical shift values with the increase in TEMPO loading, whereas the 0 ppm peak was insensitive to the radical concentration changes. It follows that the surface water was affected by TEMPO, while the structural hydroxyl groups were not. Therefore, the spectra in Figure 8 were scaled to the 0 ppm peak. As the radical concentration increased, the rotational sidebands corresponding to the main water peak became more intense. This is typical of the NMR spectra of paramagnetic materials.

In the spectra, there was also a minor signal at 1.5 ppm. Its intensity seemingly increased with the TEMPO concentration due to the growth of the underlying low-frequency wing of the water line. The origin of minor peaks between 0 and 5.5 ppm was discussed in the literature but has not been fully clarified yet. For example, they can arise from water molecules trapped inside the HA channels [46].

The most striking feature of the discussed spectra is the small signal at 16.5 ppm, which has an atypical position, probably caused by a paramagnetic shift (pseudocontact and contact), which is located on the high-frequency side of the main water signal. Moreover, this signal is broad and its intensity grows with the increase in radical concentration. Therefore, the 16.5 ppm signal may be tentatively assigned to the surface water molecules to which the radical is bound.

#### 3.3.2. HA259

**Figure 9:**
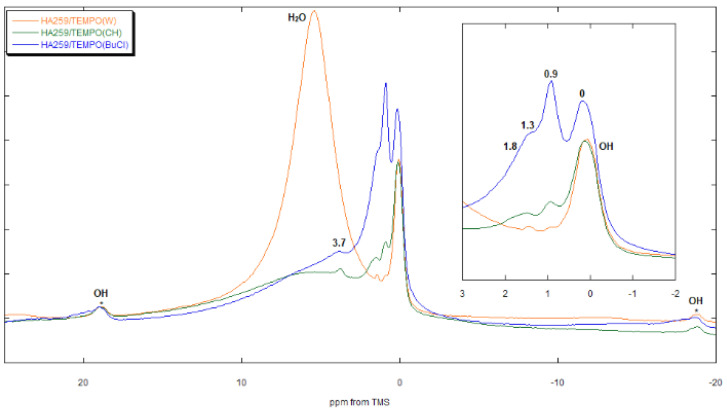
^1^H NMR spectra of the composites obtained via the TEMPO adsorption on HA259 from 0.5 mM solutions in CH, BuCl and W. The spectra were normalized to the same sample mass. Asterisks denote spinning sidebands. The inset shows a zoomed region from −2 to 3 ppm.

The spectra of the HA259/TEMPO composites (Figure 9) contain signals from structural hydroxyl groups of the apatite (0 ppm), water (5 ppm) and remains of organic solvents. The signal at 16.5 ppm detected for the HA83/TEMPO composites is absent. In the spectrum of HA259/TEMPO(BuCl), there is a set of BuCl peaks at 3.7 1.8, 1.3 and 0.9 ppm, the latter of which, a fairly intense one, overlaps with the OH^−^ peak, making it seemingly higher. In the spectrum of HA259/TEMPO(CH), there are signals from CH as well, but they are very weak. In this case, the peak at 3.8 ppm cannot be ascribed to CH because of a relatively high chemical shift. This could originate from water molecules trapped inside the apatite channels [46] or from the crystal surface P-OH groups [47]. In the case of HA259/TEMPO(BuCl), the peak is probably overlapped with the -CH_2_Cl peak of 1-chlorobutane.

HA259 was anhydrous. However, because the proton NMR is very sensitive, even formally anhydrous apatite can give a weak signal from residual water. In the case of HA259/TEMPO composites prepared using organic solvents, this faint water signal was present at about 5 ppm. This was further reduced through the adsorption of small amounts of TEMPO, so in the spectra of the composites prepared from 0.5 mM solutions of TEMPO in CH or BuCl, the water signal is indeed very weak (Figure 9). Of course, dehydrated apatite readily soaks up water vapor from the air, so the contaminating water could also have been introduced if the composites came into contact with air during measurements. Another possible source of water in the composites is residual water from organic solvents. Naturally, for HA259/TEMPO(W) the water signal was very intense, with its chemical shift being practically independent of the radical loading.

## 4. Discussion

### 4.1. Adsorption Mechanisms

Retainment of the paramagnetic character of TEMPO meant that the >NO· groups did not form covalent bonds with the HA surface [48] and that the radical was bound to hydroxyapatite either through the oxygen or the nitrogen atom, but not through both of them simultaneously, since such complexes would have been EPR-inactive [49]. The obtained EPR results showed that if the apatite was anhydrous, all the TEMPO molecules were attached directly to its surface (HA259, the lone component 1 of the EPR spectrum). If the apatite was hydrated, over 80% of the radical molecules were attached directly to the apatite surface (HA83, component 1 of the EPR spectrum, see Table S2). This means that TEMPO effectively competed with water for the adsorption sites in HA83. Therefore, it is necessary to identify the main adsorption sites of the applied apatites.

Regarding the anhydrous HA surface, there are two possible adsorption sites: Ca^2+^ ions and P-OH groups. Since for a given solvent the K_L_ values of fitted Langmuir isotherms were similar for the two HAs, it is reasonable to assume that TEMPO chose the same adsorption site for both HA83 and HA259. The assumption is also supported by the fact that after adsorption from CH on HA83 previously dried at 200 °C, the EPR signal shape resembled the one after adsorption on the anhydrous HA259. This also justifies using the same **A** and **g** tensor values of component 1 for the two hydroxyapatites.

The EPR spectra of the composites containing HA259 consisted of one component only. This indicated that only one kind of adsorption site was occupied and that it contained Ca^2+^ cations, because there are several types of P-OH groups in apatites [50] and they would have probably given different signals. The concept of Ca^2+^ ions being the adsorption site for TEMPO is also supported by the study by Lunina et al. [51], who showed that for nitroxide radicals, coordinately unsaturated metal cations, due to higher Lewis acidity, are stronger adsorption centers than surface hydroxyl groups.

Therefore, we infer that the radical is linked to the Ca^2+^ adsorption site by means of its oxygen atom. This seems most probable for several reasons. First, the nitrogen atom of TEMPO should be less accessible because of the steric hindrance created by the four neighboring methyl groups. Second, our computer simulations favor the 90° angle of the >N−O∙ axis to the apatite surface, particularly for lower and moderate initial TEMPO concentrations, when the radical has enough freedom to take an unstrained orientation. With the adsorption through the TEMPO nitrogen atom, this angle would presumably be much smaller, because the nitroxide group axis and the piperidine ring of TEMPO would have to be almost parallel to the adsorbent surface (cf. the analogy to the Al^3+…^ N−O∙ case [49]). Third, the coordination of the TEMPO oxygen atom to a cation should increase the spin density on the nitrogen atom and markedly increase the A_zz_ component of the ^14^N hyperfine coupling tensor **A**, which is indeed the case

If hydroxyapatite contains surface water, the water molecules are preferentially coordinated to the surface Ca^2+^ cations at a 1:1 ratio. It was also shown that there are approximately 4 calcium cations per 1 nm^2^ of the HA surface [52]. According to our calculations for HA83, based on the amount of surface water obtained from TGA-DSC analysis, each calcium cation on the surface of this hydroxyapatite was occupied by a water molecule, so a water monolayer was formed. It follows that TEMPO had to compel a number of water molecules to leave their Ca^2+^ adsorption sites. In proton MAS NMR, water usually gives a large signal, which is common for water molecules from different environments and having an averaged chemical shift. For HA83/TEMPO(CH) and –(BuCl), the main water signal appeared at about 5.5 ppm (Figure 8), with contributions from both the adsorbed and displaced water molecules. The former had a higher chemical shift than the latter, because they were subjected to electron polarization by the Ca^2+^ cations (the more acidic the protons, the higher their chemical shift). Furthermore, it is likely that the displaced water molecules formed small associates (dimers, trimers, etc.) by means of intermolecular hydrogen bonds. Compared to bulk water, such multimers have a significantly decreased chemical shift, i.e., well below 5 ppm. Both effects, the electron polarization of the adsorbed water and the possible association of the displaced water, would explain the stepwise motion of the common water signal towards a lower chemical shift with the increase in TEMPO loading (Figure 8). Moreover, the displaced water molecules probably gathered somewhere near the radical molecules on the HA83 surface, so the common water signal also experienced paramagnetic broadening, which in turn caused a seeming decrease in its intensity. The above discussion strongly supports the concept that the Ca^2+^ cations are the main surface adsorption sites for TEMPO molecules. This enforces the assignment of the EPR component 1 to the radical molecules bound to calcium cations.

We presume that some TEMPO molecules form hydrogen bonds with the surface water molecules bound to Ca^2+^ ions instead of replacing them. The protons of such water molecules are expected to be more acidic than the protons of bulk water due to an electron density shift towards the cations. Therefore, those water molecules are more amenable than regular water molecules to the formation of hydrogen bonds (HBs) with TEMPO.

The TEMPO adsorption process mediated by the surface water has already been linked to the EPR component 2 and the proton NMR peak at 16.5 ppm (cf. respective Results sections). This assignment of the magnetic resonance signals is justified, because both are present in the spectra of hydrated materials (HA83/TEMPO) and missing from the spectra of anhydrous materials (HA259/TEMPO) prepared by TEMPO adsorption from organic solvents. The unusually remote high-frequency position of the NMR signal of water bound to TEMPO via HBs can be explained by a paramagnetic shift, which is consistent with the discussed adsorption mechanism. Paramagnetic contact shifts caused by TEMPO have already been reported in the literature, e.g., by Kolodziejski et al. [53,54,55]. In addition, the relatively high values of the EPR parameters <A> and A_zz_ of component 2 (Table 4), the moderate mobility of pertaining TEMPO molecules and the isotropic-like manner of their motion are also in accordance with the discussed TEMPO adsorption by means of surface water.

The adsorption mediated by the surface water was probably less preferred by the radical, because the EPR component 2 and the NMR signal at 16.5 ppm were both minor in their spectra of the respective resonances. The contribution of this mechanism to the overall TEMPO adsorption on HA83 was dependent on the radical concentration in the CH and BuCl adsorption solutions, which never exceeded 21%, as calculated for component 2 of the EPR spectra (Table S2).

To sum up, the TEMPO radical competed with water for the Ca^2+^ adsorption sites, occupying them when it succeeded or forming HB with the adsorbed water if it failed. The competition was won in about 80% cases. It should be noted that 1-chlorobutane can also participate in the adsorption process on HA259. This was confirmed by the quite large signals of BuCl in the NMR spectra of the HA259/TEMPO materials (Figure 9). Curiously enough, the BuCl signals were absent in the spectra of the materials containing HA83, meaning that the presence of water influences this process, preventing BuCl from adsorbing on the apatite.

### 4.2. Distribution and Dynamics of TEMPO on Hydroxyapatite

Despite the limitations of our computational approach, it is clearly visible that irrespective of the hydroxyapatite and solvent, even for the lowest initial radical concentrations, the TEMPO molecules located directly on the HA surface were very close to one another. Here, we would like to emphasize that for HA259/TEMPO, the calculated mean interspin distances fall in the approximate range of 1.6–1.0 nm and that the TEMPO molecule diameter perpendicular to the N−O· axis equals 0.73 nm. The shortest distances between the surface Ca^2+^ cations in the hexagonal HA lattice are 0.41, 0.42, 0.58 and 0.63 nm ([56], p. 225). It follows that the TEMPO molecules cannot be attached to the directly neighboring Ca^2+^ cations. Nevertheless, the interspin distance of 1.6 nm, calculated for the lowest TEMPO loadings on HA259, is still unexpectedly short. This close proximity of the TEMPO molecules adsorbed on Ca^2+^ was also confirmed by the spectral shapes and fitted EPR parameters. In particular, apart from the two initial data points after adsorption from BuCl on HA259, the occurrence of spin exchange and its influence on the EPR component 1 was detected for all the studied composite samples. This is important information on the distances between the TEMPO molecules adsorbed on calcium cations, because the spin exchange is observed when paramagnetic centers collide with each other or are in very close proximity (usually below 1.5 nm), so that the orbitals containing unpaired electrons can overlap [38].

These findings, considered together with the maximum values of the HA surface area available per one TEMPO molecule (Table 3), led us to the assumption that the radical adsorbed on the Ca^2+^ cations was not distributed evenly on the HA surface but rather formed patches on it. Such patch-wise adsorption has already been described for nanoparticles [57]. Probably at the highest TEMPO loadings the distribution became more homogenous, especially in the case of adsorption from CH on HA83.

Since it was not possible to fit the oss parameter for the radical molecules forming hydrogen bonds with the surface water of HA in the HA83/TEMPO(CH) and HA83/TEMPO(BuCl) composites, we calculated and analyzed the ∆H_pp_ linewidths of the EPR component 2 after it became a single line dominated by SE (Table S3). The conclusion was that in the case of the TEMPO molecules interacting with the HA surface water, the dipole–dipole interactions were present while the Heisenberg spin exchange was negligible, probably due to the fact that there were less radical molecules in this adsorption environment and that they were further away from one another.

Regarding the mobility of TEMPO molecules, they behaved rather logically; that is, the mobility decreased with the increase in radical loading because of radical crowding. Since the dynamics of the adsorbed TEMPO is not a topic of prime interest for a material scientist, we address it more precisely in the Supplementary Materials. Here, we only state that in this case it is very important how the TEMPO nitrogen atom uses its LPs during adsorption. Simple geometrical reasons combined with considerations of how the electric potential around the adsorption site is influenced by the radical rotation imply that for β_D_ = 45°, Ca^2+^ interacts with only one of the electron lone pairs on the TEMPO oxygen atom, while β_D_ = 90° interacts with both of them at the same time. It follows that for HA83 the former coordination mode prevails, while for HA259 the latter mode dominates.

### 4.3. The Role of a Solvent

From a biomaterial standpoint, when choosing a solvent, one should consider not only the amount of the radical that can be adsorbed on hydroxyapatite but also the toxicity of the solvent and the possibility of its presence in a composite after adsorption.

As shown by the spectrophotometric and EPR results, there is a decrease in TEMPO adsorption with an increase in solvent polarity. A less polar solvent is better during adsorption because of its weaker interaction with TEMPO, leading to an easier adsorption of the radical on HA. Furthermore, a polar solvent is harder to remove from the apatite surface, meaning it can block adsorption sites to some extent, making them unavailable for TEMPO. Therefore, by controlling the solvent polarity and the concentration of the initial TEMPO solution, one can adjust the radical loading.

Regarding the toxicity, water was the best of our solvents. Unfortunately, it was the worst for the TEMPO adsorption process. Here, we should point out two different cases: (1) when water formed a monolayer on the apatite surface and bulk water was not present during the adsorption, performed from organic solvents (HA83); (2) when bulk water was introduced during the adsorption from aqueous TEMPO solutions and deposited on the apatite surface in several aqueous adsorption layers (both HA83 and HA259). In case (1), TEMPO was able to compete quite effectively with water for the Ca^2+^ adsorption sites and also to form hydrogen bonds with water molecules coordinated to the Ca^2+^ cations. However, the TEMPO adsorption was in that case much less efficient than on an anhydrous HA surface (when comparing the intensities in Figure 2b and Figure 3a). In case (2), the amount of water in the adsorption environment was extremely high. Since water has higher affinity towards HAs than TEMPO, it thoroughly blocked the Ca^2+^ adsorption sites. This, in turn, caused TEMPO to attach either to the water molecules coordinated directly to the cations (EPR component 2) or to relatively mobile water clusters located either on the apatite surface or in the spaces between apatite grains (EPR component 3). However, the water molecules coordinated to Ca^2+^ cations were utilized to a large extent to form hydrogen bonds with excessive water molecules. Therefore, the adsorption of TEMPO on those sites was significantly limited as well. This explains why the EPR spectra of HA259/TEMPO(W) and HA83/TEMPO(W) were generally very weak (Table S1). One may also argue that in aqueous solutions water can form shells enclosing TEMPO, leading to a significantly reduced local polarity at the nitroxide [58] and hindering its adsorption.

Apart from the expected competition with TEMPO for the HA surface, dependent on the solvent polarity, the choice of solvent seemed to affect the distribution of the radical between the two main adsorption environments present on HA83, e.g., at the lowest initial TEMPO concentrations the contribution of component 2 was higher for BuCl than CH. Additionally, the A parameter values (Table 4) for component 2, being different than those of component 1, suggested that the solvent could be present on HA after adsorption. The contamination of the HA83 surface with BuCl was directly confirmed by the proton NMR spectra of the HA83/TEMPO(BuCl) materials (Figure 9). The presence of BuCl could influence the polarity around surface water molecules to which the radical was attached and could affect the adsorption equilibria. Moreover, it was shown that in solutions, nitroxides can form halogen bonds with molecules containing iodine atoms [59] Analogous bonds can be envisaged with the chlorine atom of BuCl.

Cyclohexane seems to be the most promising of the three applied solvents. It affords the best TEMPO loadings and it does not adsorb on apatites, so it does not contaminate the HA/TEMPO composites. Of course, the study should be extended to other solvents to look for even better alternatives for TEMPO adsorption on apatites.

### 4.4. Comparison of the Hydroxyapatites

The two studied HAs differed with respect to their SSA, crystal size and hydration degree. For the radical adsorbed directly on the HA surface, adsorption sites were the same for both apatites–the radical attached to the surface Ca^2+^ cations. The affinity levels of TEMPO towards the two HAs were quite similar for a given solvent, as evidenced by the K_L_ values presented in Table 2. In spite of this, the Q_0_ value ratios of HA259 to HA83 for organic solvents did not correspond to the SSA ratio of hydroxyapatites—the Q_0_ ratios equaled 4.93 and 8.35 for CH and BuCl, respectively, while the SSA ratio amounted to 3.10. Those discrepancies could stem from the presence of water or lack thereof, as well as from the potentially different affinity levels of solvents to the studied hydroxyapatites.

However, comparing the two HAs in such a direct manner could be misleading, since there are three main types of surfaces in hydroxyapatite crystals: (001), (101) and (010). The last of these can have three different terminations of crystal faces: stoichiometric (-OH terminated), Ca-rich (Ca-exposed) and P-rich (PO_4_-exposed) [60,61]. According to previous studies, water interacts differently with each of the mentioned surfaces, which leads us to believe that the same will be true for TEMPO. Since the >NO· group of the radical is shielded by methyl groups, the radical molecules should predominantly choose the Ca-rich environment for adsorption. So far there has been little information concerning the occurrence frequency of each termination in various hydroxyapatites. Therefore, it should not be surprising that the amount of adsorbed TEMPO was not proportional to the SSA, especially considering the different synthesis methods and crystal morphologies and the fact that the SSAs were determined through the adsorption of nitrogen, which interacts with HAs in a different manner than water or TEMPO.

The crystal size of apatites controls not only their SSA, but also affects the macrostructure of their powders and the surface structure of their crystals. Our apatites had substantially different crystal sizes, because crystals of HA83 are, on average, about six times larger than those of HA259. The apatite powders are composed of separate crystals and their grains. Between those particles there are spaces of various sizes, which can take on water during TEMPO adsorption from this solvent. It is expected that such spaces are, on average, larger in HA83 than HA259, and that they can admit water, which would remain there in a semiliquid state. The TEMPO molecules occupying such compartments would experience rather isotropic motion and give quite sharp triplets in EPR, as seen in Figure 6. It follows that those triplets should be more intense for HA83 than HA259, which is indeed so (Table S1). One should also take into account that the HA259 crystals are extremely small. We found that the average dimensions of their projections onto the TEM picture plane were 19.1 nm × 8.5 nm. At the same time, the parameters of the hexagonal crystallographic unit cell were a = b = 0.943 nm and c = 0.688 nm [62]. It follows that such crystals can accommodate only several unit cells across their shortest dimensions, so a disorder in the crystal interior would have an impact on the crystal surface structure, and vice versa. Thus, when discussing the apatite surface structure one should consider both the different terminations of the crystal faces and the structural implications of the small crystal dimensions.

The hydration degree of the apatites was also crucial, as was discussed before. HA259 was anhydrous, while HA83 contained a monolayer of surface water. This had a great influence on the adsorption sites offered by HA and their availability to TEMPO.

### 4.5. EPR Signal Intensities Versus Adsorption Isotherms

The discrepancy between adsorption isotherms and EPR signal intensities, especially being well visible for HA83 but also present to a lesser degree for HA259, is a complex problem. Assuming that the spectrophotometric and EPR measurements were accurate, there are two possibilities: either some of the adsorbed TEMPO molecules were not observed by EPR or the final amount of the radical measured directly in the composite (EPR) was different from the amount measured indirectly on the basis of TEMPO lost from the adsorption solution (spectrophotometry). We believe that the second explanation would not be able to account for such large differences between those experimental approaches. Therefore, we opted for the first option.

We found three reasons which could explain the loss of paramagnetic properties: the formation of the so called “EPR-inactive” complexes, described by Katter et al. [49], the dimerization of TEMPO [63] and the disproportionation of the radical molecules represented by component 2 in the EPR spectra. The disproportionation can take place due to the higher acidity of the hydroxyl groups of water molecules bound to the surface Ca^2+^ cations [64]. A similar phenomenon was observed for TEMPO interacting with acidic centers in B_2_O_3_–Al_2_O_3_ systems [65].

In our opinion, the most probable explanation for the discussed issue entails the formation of EPR-inactive complexes and the radical disproportionation pf HA83, while for HA259 only the EPR-inactive complexes can be taken into consideration. A more detailed discussion of this topic is available in the Supplementary Materials.

### 4.6. Potential Applications of the Obtained Composites

The adsorption method used by us gave reproducible results for organic solvents. The obtained composites were fairly stable during the studied time period, if stored under appropriate conditions, which means that these materials can be further studied, modified and used. There are two general fields where the hydroxyapatite/TEMPO composites could potentially be applied: physicochemical and medical areas.

When it comes to physicochemical applications, it seems that TEMPO could indeed be used as a spin probe to study certain properties of the hydroxyapatite surface using EPR. Our work showed that the EPR spectra can definitely disclose some information regarding the presence and distribution of water on the HA surface, as well as the acidity of adsorption sites. As we used stoichiometric, unsubstituted HAs, it was not possible to determine whether the radical would be suitable for detecting ionic composition changes in the hydroxyapatite surface. However, based on the studies describing the interactions of TEMPO and zeolites substituted with various ions, we consider it plausible [29,32]. The same could potentially be true for the adsorption of biologically active compounds on hydroxyapatites. It is worth noting that only composites containing small TEMPO amounts could be used in such studies. Otherwise, very little information would be obtained from the EPR spectrum, since it would be a broad singlet.

Additionally, there is a possibility that the materials containing nitroxide radicals and hydroxyapatite will be useful as metal-free contrast agents in MR imaging of hard tissues. This is still a challenging field, because bones and enamel contain relatively few protons and the transverse relaxation times in those tissues are short [66]. The utility of nitroxides in MRI has already been proven. There are, of course, some limitations to their use as contrasts, but it has been shown that they can be overcome, e.g., through chemical modification of the radicals and by linking them with organic polymers [67].

Since both TEMPO and hydroxyapatites can be used as catalysts, it is possible that the combined material will also have this application. Nitroxide radicals and compounds modified by them are mainly used in oxidation reactions [68,69], while hydroxyapatites can be employed in numerous processes, e.g., racemization, biogasoline synthesis and reactions requiring transition metal catalysts [70]. The issue of whether the HA/TEMPO composites will have additional benefits as catalysts requires further investigation.

Regarding the medical aspects, the composites could potentially be applied as antioxidative and anticancer fillers after bone surgery. Of course, for such cases many additional requirements would have to be met beforehand. An appropriate initial radical concentration would have to be chosen along with the radical itself; it is possible that other nitroxides, e.g., 4-OH-TEMPO would prove to be better at body temperature, as TEMPO can undergo sublimation even at RT. Additionally, an appropriate solvent would have to be chosen, considering its polarity, affinity to the HA and toxicity. From the three studied solvents, cyclohexane seems to be the best choice, since it allows the adsorption of reasonably high radical amounts and is not present in the dried composites due to its low polarity and high volatility. To achieve the best repeatability and stability, it would probably be best to use hydroxyapatites of high SSA, obtained via a traditional wet method, since the amount of water in such materials would not change upon contact with air. The materials would need to undergo all of the necessary biological tests. Assessments of the cyto- and genotoxicity of the HA/TEMPO and HA/4-OH-TEMPO materials as well as the preliminary evaluation of their anticancer activity in vitro are currently in progress.

## 5. Conclusions

The main conclusions of this work can be summarized as follows:

- It is possible to reproducibly adsorb the TEMPO radical on hydroxyapatite, even if it contains a monolayer of surface water;
- After adsorption, TEMPO did not lose its paramagnetic character. The EPR signal shape changed significantly, indicating a decrease in the radical mobility with respect to the solution;
- The main factors influencing the radical loading are the solvent polarity and hydroxyapatite surface area. The more polar the solvent is, the more it competes with the radical, thereby diminishing its adsorption;
- In all studied cases, the adsorption was limited to a monolayer of TEMPO, and where it was possible to fit model isotherms, the best fit was achieved with the Langmuir model. The similar values of the Langmuir constant indicate that the affinity of TEMPO towards both apatites and the mechanisms of adsorption from organic solvents were the same;
- In the case of the adsorption from organic solvents, adsorption sites available to the radical were dependent on whether the apatite was hydrated or not:
  ο On the anhydrous HA259, all radical molecules were linked directly to the HA surface, presumably through the coordination bond between the TEMPO oxygen atom and the hydroxyapatite surface calcium cations;
  ο On the hydrated HA83, the majority of the TEMPO molecules also coordinated directly to the surface calcium cations, thereby displacing surface water molecules. Some TEMPO molecules (less than 20%), however, were bound through the hydrogen bonds of their oxygen atoms to the not yet displaced surface water molecules and were consequently more mobile than the directly adsorbed TEMPO molecules.
- For the adsorption from water, the distribution of TEMPO on the apatite surface was different than after adsorption from organic solvents and was similar for both HAs. As all the surface calcium cations were occupied by water, the majority of the radical molecules were linked to those surface water molecules via the N−O·^…^H−O hydrogen bonds, while the rest presumably formed such hydrogen bonds with water clusters located in spaces between particles of the apatite powder or with water molecules in the outer layers, further away from the HA surface;
- The analysis of dipole–dipole interactions and spin exchange allowed us to estimate mean distances between the TEMPO molecules. This led us to the assumption that the radical was not distributed homogenously on the HA surface but rather adsorbed preferentially in patches;
- The analysis of integral intensities of the EPR signals suggested that when the crowding of the radical molecules was high, they had a tendency to interact with the calcium cations on the HA surface by means of only one lone electron pair of the nitroxide oxygen atom. Furthermore, a certain part of them was not detected by the EPR spectroscopy, either because of EPR-inactive complexes formed with the apatite surface by means of both O and N of TEMPO (for both HA259 and HA83) or because of the radical disproportionation due to the acidic character of the hydroxyl groups of water bound to TEMPO by means of the hydrogen bonds (for HA83 only);
- The obtained HA/TEMPO composites were fairly stable over one year if they were stored under appropriate conditions.

This work is worth continuing in order to develop unique hydroxyapatites with paramagnetic surfaces, which are of physicochemical and medical interest. The HA/TEMPO composites should be examined in terms of their cytotoxicity and genotoxicity. The experimental study should be extended to other apatites and nitroxide radicals and to a wider range of solvents. Theoretical calculations must be performed to support the experimental work and to explain the currently unsolved problems.

## Figures and Tables

**Table 1:** Fit accuracy comparison for the Langmuir and Freundlich models.

Solvent	R^2^_L_	R^2^_F_	χ^2^_L_(×10^−5^)	χ^2^_F_(×10^−5^)	ΔAIC_c_^a^	Evidence Ratio L/F ^b^
**HA83**						
**CH**	0.979	0.946	3.77	9.85	16.32	3498
**BuCl**	0.910	0.898	0.79	9.02	2.21	3
**HA259**						
**CH**	0.992	0.986	37.58	64.59	9.20	99
**BuCl**	0.996	0.990	1.96	4.66	14.72	1572

^a^ The absolute difference between the AIC_c_ scores of the Langmuir and Freundlich fitted isotherms. ^b^ The evidence ratio is equal to $1/e^{-0.5\;\Delta AIC_{c}}$. It shows how many times the Langmuir model is more likely to be correct than the Freundlich model.

**Table 2:** Parameters of the fitted Langmuir isotherms and TEMPO molar absorption coefficients for the studied solvents.

Solvent	HA83		HA259		ε [L·mol^−1^·cm^−1^]
	Q_0_ [mmol·g^−1^]	K_L_ [L·mmol^−1^]	Q_0_ [mmol·g^−1^]	K_L_ [L·mmol^−1^]	
CH	0.0493 ± 0.0014	0.351 ± 0.036	0.243 ± 0.006	0.292 ± 0.021	1836
BuCl	0.0139 ± 0.0030	0.072 ± 0.028	0.116 ± 0.006	0.061 ± 0.005	1922
W	-	-	-	-	2060

**Table 3:** Surface area of hydroxyapatite per one TEMPO molecule (nm^2^) for the chosen experimental data points (c_0_) and for the calculated maximum adsorption capacity values (Q_0_).

Q_0_		CH		BuCl	
C_0_		HA83	HA259	HA83	HA259
**c** **_0_** **[mM]**	0.5	19.47	17.87	286.01	124.89
	5	4.35	3.50	41.87	16.54
	14	3.35	2.24	22.32	8.08
**Q_0_**		2.81	1.77	9.95	3.71

**Table 4:** EPR parameters of TEMPO for its composites containing HA259 and HA83, reported for separate spectral components. The parameters were computed using the MultiComponent program. The **g** tensor was precisely determined for 0.5 mM HA259/TEMPO(BuCl) and was kept the same for all fittings: g_ii_ = 2.0058, 2.0047 and 2.0014. Concentration ranges of TEMPO (in mM) for variable parameters are given in the square brackets below their extreme values. Component 3 for HA83/TEMPO(CH) and HA83/TEMPO(BuCl) was only detected for the TEMPO concentrations specified in the table for those composites.

Apatite	HA259	HA83	HA83	HA83	HA259	HA83	HA83	HA83	HA259	HA259	HA83	HA83
Solvent	CH	CH	CH	CH	BuCl	BuCl	BuCl	BuCl	W	W	W	W
Component	1	1	2	3	1	1	2	3	2	3	2	3
Contribution (%)												
	100	99.0–79.4 [0.5–16]	1.1–20.6 [0.5–16]	0.07–0.04 [1–4]	100	98.0–80.0 [0.5–16]	2.0–20.0 [0.5–16]	0.06–0.04 [2–3]	94.2 [8] ^1^	5.8 [8] ^1^	89.8 [8] ^1^	10.2 [8] ^1^
Nitrogen-14 hyperfine coupling tensor												
A_xx_ (G)	7.72	7.72	5.69		7.72	7.72	7.93		9.17	9.17	8.82	8.82
A_yy_ (G)	8.48	8.48	5.69		8.48	8.48	7.93		8.83	8.83	8.60	8.60
A_zz_ (G)	37.70	37.70	38.85		37.70	37.70	34.54		33.10	33.10	33.63	33.63
A_iso_ (G)				17.28				17.28				
Line broadening, dynamic parameters and tilt angle												
W_iso_ (G)	9.8–14.4 [0.5–16]	14.4–16.7 [0.5–16]	4.3–21.8 [0.5–16]	3.0 [1–4]	7.8–14.2 [0.5–16]	14.4–17.3 [0.5–16]	3.0–22.0 [0.5–16]	3.0 [2–3]	18.1 [8] ^1^	1.8 [8] ^1^	14.9 [8] ^1^	1.5 [8] ^1^
ν_ex_ (10^7^ s^−1^)	5.1–30.9 [0.5–16]	30.2–246 [0.5–16]	0 [0.5–16] ^2^	0 [1–4] ^2^	0.5–14.8 [2–16]	15.5–224 [0.5–16]	0 [0.5–16] ^2^	0 [2–3] ^2^	0 [8] ^1^	0 [8] ^1^	0 [8] ^1^	0 [8] ^1^
τ_z_ (ns)	3.5–11.8 [0.5–4] ^3,4^	0.06–0.24 [0.5–3] ^3,5^			3.8–17.5 [0.5–15] ^3,5^	0.06–0.44 [0.5–6] ^3,5^						
τ_iso_ (ns)			0.54 [0.5] ^5^	<0.01 [1–4] ^6^			0.97–1.18 [0.5–1] ^5^	<0.01 [2–3] ^6^	0.3 [8] ^1,6^	0.5 [8] ^1,6^	0.2 [8] ^1,6^	0.7 [8] ^1,6^
β_D_ (degrees)	90 [0.5–16]	54 [0.5–16]	0 ^7^	0 ^7^	90 [0.5–16]	54 [0.5–16]	0 ^7^	0 ^7^	0 ^7^	0 ^7^	0 ^7^	0 ^7^

^1^ Determined only for this single concentration because other spectra were of too low quality. ^2^ Fittings insensitive to ν_ex_ for the studied concentrations. ^3^ Calculated from the R_zz_ component of the rotational tensor **R**, no rotation around the x and y axes assumed (R_xx_ = R_yy_ = 0). ^4^ For the TEMPO concentration over 4 mM a typical rigid case was found: τ_z_ > 1000 ns. ^5^ For the higher TEMPO concentrations the parameter was fixed at the latest reliable value. ^6^ Approximate values. ^7^ Fittings insensitive to β_D_ for the studied concentrations.

## Data Availability

Data are partially available in the article and in the Supplementary Materials. The rest are available on request from the corresponding author.

## Supplementary Materials

The following are available online at https://www.mdpi.com/article/10.3390/ma15062043/s1: Figure S1: DSC-TGA results for HA83. Figure S2: UV–Vis spectra of TEMPO in the studied solvents. Figure S3: TEM images: (**a**) HA83; (**b**) HA259. Figure S4: Relationships between the surface area of hydroxyapatite per one TEMPO molecule (nm^2^) and the initial TEMPO concentration (mM) in the adsorption solution. Figure S5: Relationships between the fitted EPR parameters of the HA83/TEMPO and HA259/TEMPO composites and the initial TEMPO concentration (mM) in the adsorption solution. The results were obtained using the MultiComponent computer program. Figure S6: Orientation of the coordinate system axes (CSAs) of the magnetic and rotational tensors of TEMPO (**a**–**c**) and the possible orientations of the TEMPO molecule with regard to the surface Ca^2+^ cations of HA259 (**d**,**e**) and HA83 (**f**,**g**) under experimental tilt angles β_D_ of 90° and 54°, respectively. Table S1: Integral intensities of the EPR spectra, scaled to the same sample mass. Table S2: Percentage contributions of the EPR spectral components for HA83/TEMPO composites prepared by TEMPO adsorption from 1-chlorobutane and cyclohexane. Table S3: Peak-to-peak linewidths, ∆H_pp_ in [G], of the two main EPR components fitted for the HA83/TEMPO(BuCl) and HA83/TEMPO(CH) composites. Table S4: Mean distances (nm) between paramagnetic centers of the TEMPO molecules adsorbed on HA259 (component 1), calculated using the ΔH_pp_ linewidth of the TEMPO triplet central line (method I) and the d_1_/d parameter (method II). Detailed issue description 1: Dynamics of TEMPO adsorbed on hydroxyapatite. Detailed issue description 2: EPR signal intensities versus adsorption isotherms. Procedure description: Calculations of distances between paramagnetic centers of TEMPO molecules.
