# Supplementary material for: Composites Containing Nanohydroxyapatites and a Stable TEMPO Radical: Preparation and Characterization Using Spectrophotometry, EPR and 1H MAS NMR
Source: Materials (Basel). 2022 Mar 10;15(6):2043. doi: 10.3390/ma15062043 (PMC8952365; doi:10.3390/ma15062043)
Supplement: Supplementary file 1 [file materials-15-02043-s001.zip › materials-1604793-supplementary.pdf]

materials

SUPPLEMENTARY MATERIAL

Composites containing nanohydroxyapatites and a stable TEMPO radical: preparation and characterization using spectrophotometry, EPR and ^{1}H MAS NMR

Natalia Byra [1,*], Sylwester Krukowski [1], Jaroslaw Sadlo [2] and Waclaw Kolodziejski [1]

[1] Chair of Analytical Chemistry and Biomaterials, Department of Analytical Chemistry, Medical University of Warsaw, Banacha 1, 02-097 Warsaw, Poland
[2] Institute of Nuclear Chemistry and Technology, Dorodna 16, 03-195 Warsaw, Poland

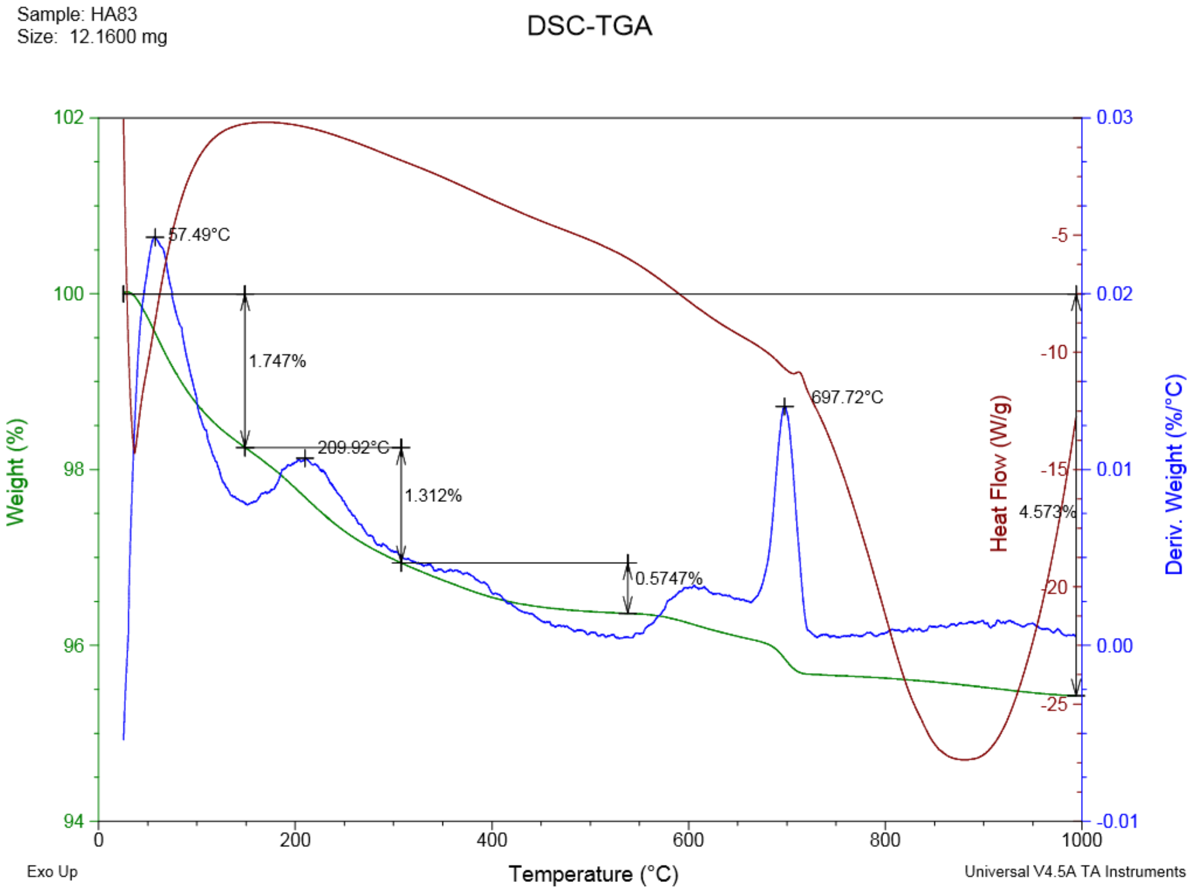

Figure S1. DSC-TGA results for HA83.

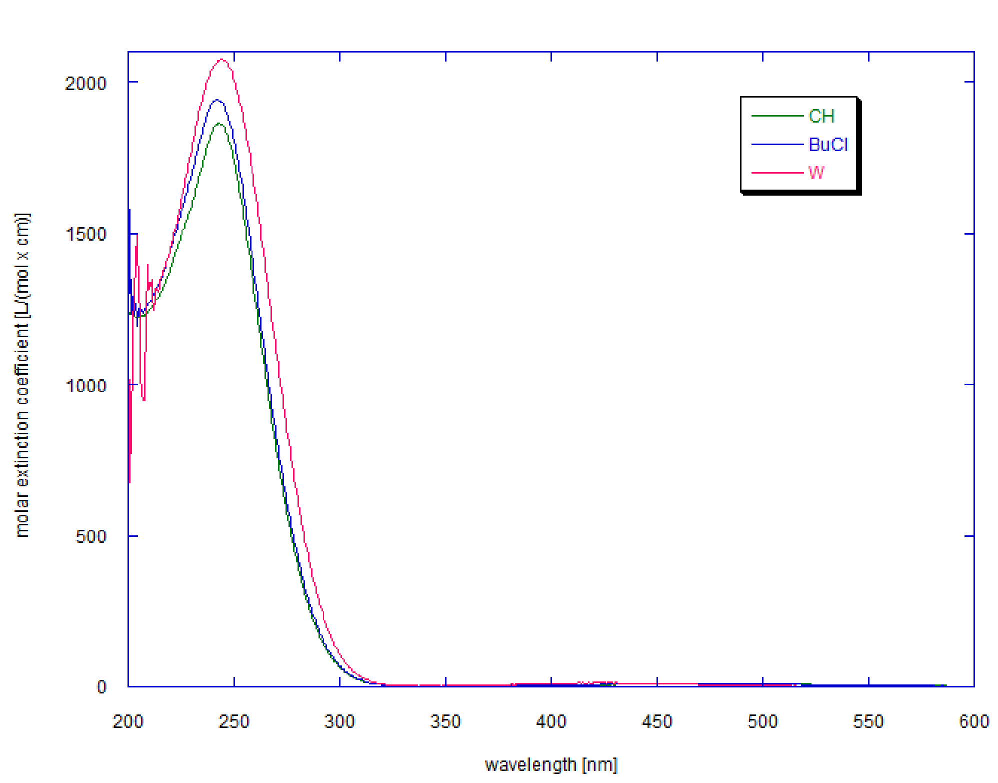

Figure S2. UV-Vis spectra of TEMPO in the studied solvents.

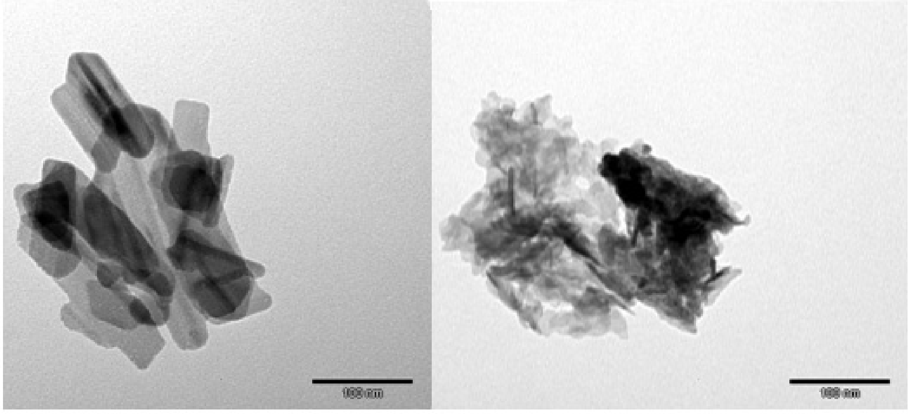

(a) (b)

Figure S3. TEM images: a) HA83; b) HA259.

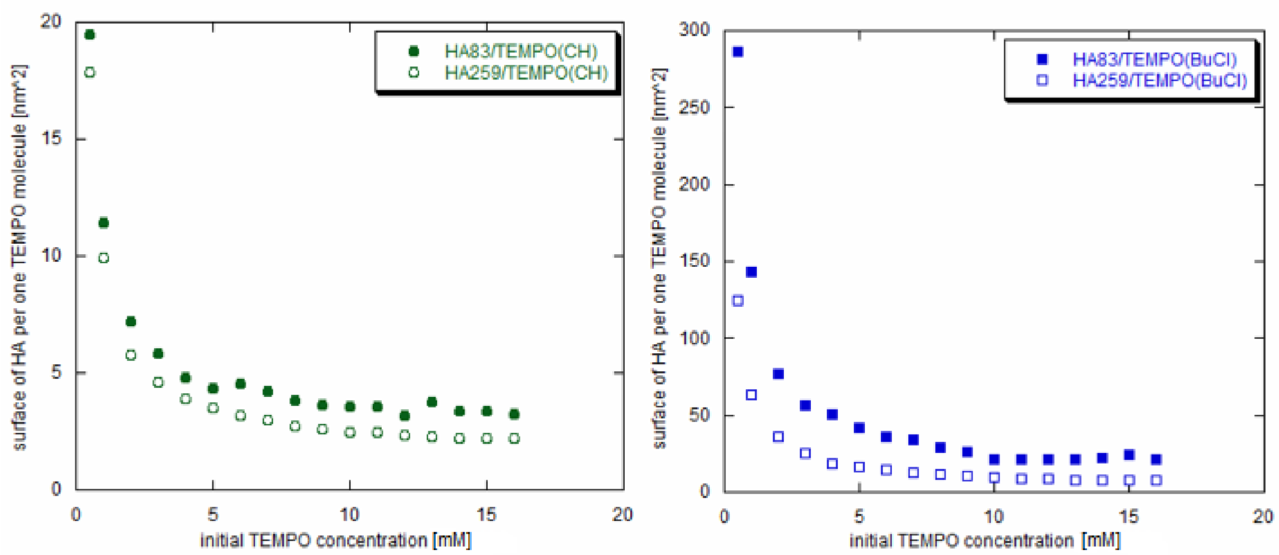

(a) (b)

Figure S4. Relationships between the surface area of hydroxyapatite per one TEMPO molecule [nm^2] and the initial TEMPO concentration [mM] in the adsorption solution: (a) after adsorption from CH; (b) after adsorption from BuCl.

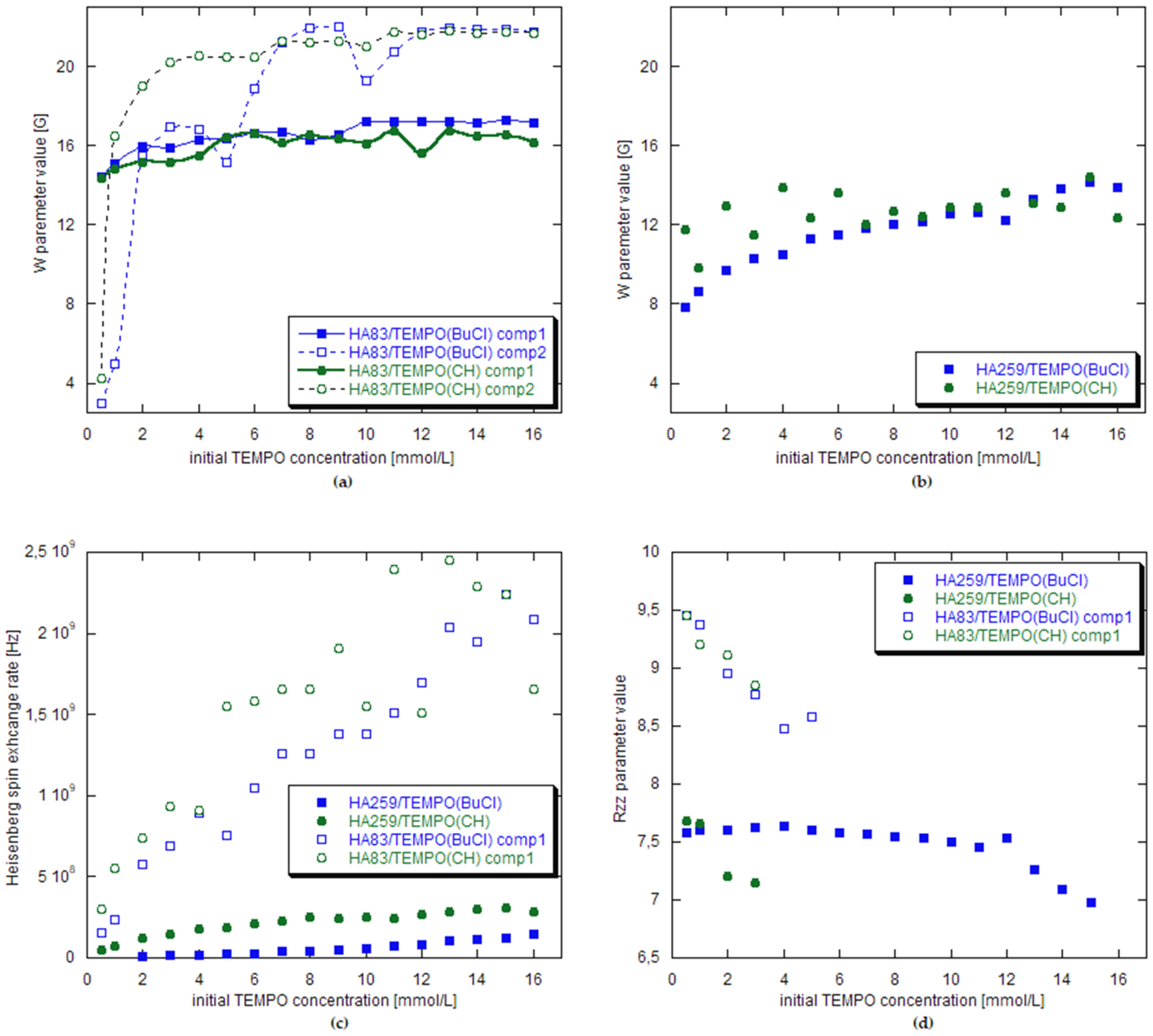

Figure S5. Relationships between the fitted EPR parameters of the HA83/TEMPO and HA259/TEMPO composites and the initial TEMPO concentration [mM] in the adsorption solution. The results were obtained using the MultiComponent computer program [1]: (a) W parameter values for HA83; (b) W parameter values for HA259; (c) spin exchange rate; (d) R_{zz} parameter values.

Dynamics of TEMPO adsorbed on hydroxyapatite

When it comes to the mobility of TEMPO molecules, it rather behaved logically, that is, it was decreasing with the increase in radical loading. Unexpected, however, was the fact that the mobility of the TEMPO molecules attached directly to the HA surface was significantly higher for HA83 than HA259 (cf. τ_z for component 1 in Table 4 in the main text). Although there were less radical molecules on HA83 than HA259, the available space was also much lower, which should have resulted in higher crowding and slower rotational diffusion. Considering the calculated mean distances between TEMPO molecules, the Heisenberg exchange rates, the W parameter values and the signal shapes obtained for both HAs, it was indeed so. Moreover, according to our EPR study, this crowding on HA83 was present from the very beginning, that is, for the lowest initial TEMPO concentrations. One explanation of this discrepancy in the mobility of TEMPO on HA83 versus HA259 could be a different tilt angle β_D: about 54° for HA83 and prevalently 90° for HA259, although in the latter case, for the highest TEMPO concentrations, convergent fittings were also possible for β_D in the 40°-70° range. Simple geometrical reasons combined with considerations of how the electric potential around the adsorption site is influenced by the radical rotation (Figure S6), imply that for β_D=45°, Ca^{2+} interacts with only one of the electron lone pairs on the TEMPO oxygen atom, while for β_D=90°, it interacts with both of them at the same time. It follows that for HA83 the former coordination mode prevails, while for HA259 the latter mode dominates.

The question is whether during the coordination of a >NO· group to a Ca^{2+} cation by means of one LP, the pertinent radical molecule has more freedom to perform rotational diffusion with preference around the *z* axis of tensor **R** than in the two LPs configuration. The problem is not trivial, because if the radical was to rotate around the *z* axis, which in the one LP case crosses the TEMPO oxygen hybrid sp^2 orbital and the Ca^{2+} cation (Figure S4 (d)), it would sweep a much larger space with its piperidine ring than in the two LPs case. One should also bear in mind that there is surface water on HA83. Therefore, close to the TEMPO molecules linked to Ca^{2+} cations there are water molecules on adjacent, other Ca^{2+} cations (if they form HBs with TEMPO, they give EPR component 2). Such water molecules might be somehow involved in the TEMPO dynamics through their presumably repulsive action against alkyl fragments of the rotating piperidine moiety and its methyl substituents. This problem may be solved using appropriate theoretical calculations, including molecular dynamics. Finally, we admit that there were some uncertainties of the EPR fittings: contrary to HA259, component 1 from TEMPO on the surface of HA83 was a single line from the beginning, which rendered the computations less accurate.

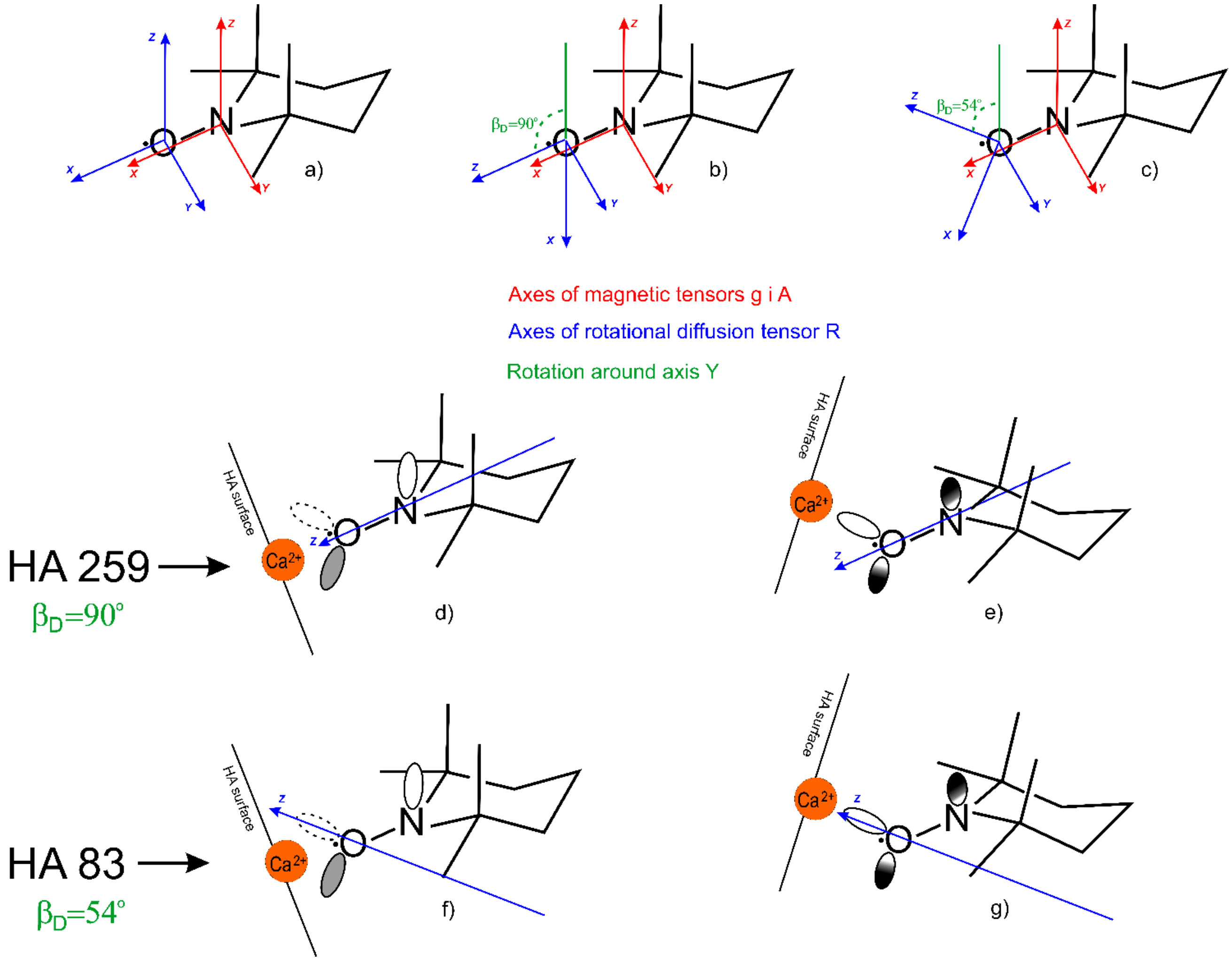

Figure S6. Orientation of the coordinate system axes (CSAs) of the magnetic and rotational tensors of TEMPO (a–c) and the possible orientations of the TEMPO molecule with regard to the surface Ca^{2+} cations of HA259 (d,e) and HA83 (f,g) under the experimental tilt angles β_D of 90° and 54°, respectively. The origins of the CSAs of the magnetic tensors **g** and **A** are traditionally placed at the nitrogen atom of the nitroxide group, while the origin of the CSA of the rotational diffusion tensor **R** is chosen at the oxygen atom of the nitroxide group, that is at the atom interacting with the HA surface Ca^{2+} cations. The preferential TEMPO rotation takes place around the z axis of the tensor **R**. In schemes (d) and (f) both oxygen atom lone electron pairs (LPs) are interacting with the Ca^{2+} cation, while in schemes (f) and (g) only one LP is interacting with the Ca^{2+} cation. During the rotation, the adsorbed TEMPO molecule is assumed to experience the least steric hindrance in the case (d) on the HA259 surface and in the case (g) on the HA83 surface.

EPR signal intensities versus adsorption isotherms

Below we discuss in detail the three possible reasons for the radical paramagnetic character loss.

One reason for this could be the so called "EPR-inactive" complexes. They are formed when a nitroxide radical is attached to the adsorbate surface via both the nitrogen and oxygen atom. This renders the radical molecule almost parallel to the surface. As has already been mentioned, in such configuration paramagnetic properties are lost, since the delocalized orbitals hosting free electron are involved in the bond formation with the adsorbent surface [2]. Considering the fact that after adsorption on HA83 from concentrated CH solutions all the TEMPO molecules are very close to one another and that the molecules can become tilted at higher crowding, this hypothesis seems plausible. Although in other cases the HA surface per one TEMPO molecule was higher, the occurrence of this phenomenon cannot be excluded, especially seeing as the differences between the amount of the radical molecules adsorbed from the two organic solvents were significantly more pronounced in the case of spectrophotometric measurements.

Another reason for the loss of paramagnetic properties could be the formation of diamagnetic dimers. It has been shown that in a solution there is an equilibrium between a radical and its dimer. Although for TEMPO the dimerization constant is relatively small due to the mentioned steric hindrance (2.5×10^{-4}), the reaction can still take place and its enthalpy in a propionitrile/butyronitrile mixture was determined to be -72.1 kJ/mol [3]. Theoretically, two types of dimers are possible in our case: S=0 dimers, where two TEMPO molecules are attached to the same Ca^{2+} cation, and S=1 dimers, where two radical molecules are adsorbed on the adjacent cations. While the former are completely devoid of paramagnetic character and, therefore, fully invisible in an EPR experiment, the latter are hard to detect in a powder sample by a classic CW-EPR experiment due to high line widths. As has already been stated, the distance between neighboring calcium cations in hydroxyapatites is between 0.41 and 0.63 nm ([4], p. 225), while the Van der Waals diameter of the TEMPO molecule amounts to 0.73 nm. It follows that the formation of the S=1 dimers on hydroxyapatites cannot take place. The S=0 dimers could potentially form, however, it would be improbable due to the steric hindrance.

The third explanation of the loss of the EPR signal intensity concerns redox properties of nitroxide radicals. As was mentioned earlier, if a water molecule is coordinated to a Ca^{2+} cation, its hydroxyl group has a pronounced acidic character and can form hydrogen bonds more easily. During the formation of the bond with TEMPO, there is a possibility of the transfer of a proton to the oxygen atom of the radical, which would cause the disproportionation of the radical and loss of its paramagnetic character [5]. Similar phenomenon was observed for TEMPO adsorbed on Al_2O_3 with an increased surface acidity [6]. Naturally, this occurrence could only apply to HA83, the hydrated hydroxyapatite. Since more radical adsorbed on HA83 from CH than BuCl, after adsorption from the less polar solvent there were consequently more radical molecules approaching water molecules coordinated to the calcium cations. This potentially lead to the disproportionation being more intense after adsorption from cyclohexane.

Table S1. Integral intensities of the EPR spectra, scaled to the same sample mass.

Initial TEMPO conc. [mM]	HA83/TEMPO			HA259/TEMPO		
	CH	BuCl	W	CH	BuCl	W
**0.5**	2285	780	5	8777	1866	0
**1**	3741	1624	7	15557	4113	0
**2**	6314	3457	26	28763	6856	25
**3**	8295	4837	50	31330	11857	8
**4**	9989	5954	51	40325	13424	224
**5**	10705	7670	61	44448	16591	16
**6**	11767	8876	65	45499	19247	24
**7**	14169	12175	61	50341	23013	27
**8**	13812	13462	73	53545	24168	64
**9**	14923	15275	74	56064	26534	60
**10**	16415	15270	79	55698	31152	36
**11**	16683	16980	90	60893	28279	32
**12**	16332	18912	84	69965	32389	56
**13**	17445	24059	65	63537	38025	39
**14**	17709	23032	71	67238	36173	76
**15**	18968	25005	86	62962	37089	47
**16**	17631	26926	97	66512	35337	44

Table S2. Percentage contributions of the EPR spectrum components for HA83/TEMPO composites prepared by TEMPO adsorption from 1-chlorobutane and cyclohexane. The results were obtained using the MultiComponent computer program [1].

Initial TEMPO concentration [mM]	1-chlorobutane			cyclohexane		
	comp. 1	comp. 2	comp. 3	comp. 1	comp. 2	comp. 3
**0.5**	97.48	2.52	-	98.95	1.05	-
**1**	97.97	2.03	-	89.94	9.99	0.07
**2**	87.47	12.47	0.06	86.92	13.03	0.05
**3**	88.11	11.85	0.04	86.70	13.26	0.04
**4**	89.03	10.97	-	86.32	13.64	0.04
**5**	89.54	10.46	-	82.35	17.65	-
**6**	85.18	14.82	-	82.84	17.16	-
**7**	82.50	17.50	-	80.67	19.33	-
**8**	80.03	19.97	-	81.73	18.27	-
**9**	81.70	18.30	-	80.84	19.16	-
**10**	90.50	9.50	-	82.07	17.93	-
**11**	88.30	11.70	-	80.21	19.79	-
**12**	85.59	14.41	-	79.41	20.59	-
**13**	84.90	15.10	-	79.87	20.13	-
**14**	85.39	14.61	-	79.51	20.49	-
**15**	84.09	15.91	-	79.73	20.27	-
**16**	85.59	14.41	-	80.96	19.04	-

Table S3. Peak-to-peak linewidths, ΔH_{pp} in [G], of the two main EPR components fitted for the HA83/TEMPO(BuCl) and HA83/TEMPO(CH) composites. The fittings were done using the MultiComponent computer program [1].

Initial TEMPO concentration [mM]	1-chlorobutane		cyclohexane	
	comp. 1	comp. 2	comp. 1	comp. 2
**0.5**	43.58	-	33.18	-
**1**	37.98	-	26.59	45.58
**2**	27.78	49.18	24.79	46.58
**3**	26.39	49.98	23.79	47.38
**4**	25.79	49.98	23.99	47.58
**5**	26.59	49.18	22.79	47.38
**6**	24.99	50.78	22.99	47.38
**7**	24.39	51.98	22.39	47.78
**8**	23.59	52.38	22.59	47.58
**9**	23.59	52.38	21.99	47.98
**10**	24.39	50.98	22.39	47.78
**11**	23.99	51.58	21.99	47.98
**12**	23.59	52.18	22.00	47.98
**13**	22.99	52.18	21.99	48.18
**14**	22.99	52.18	21.80	48.18
**15**	22.79	52.18	22.00	48.18
**16**	22.79	52.18	22.20	48.18

Calculation of distances between paramagnetic centers of TEMPO molecules

To gain more insight into the radical distribution on the HA surface, we estimated mean distances (x) between TEMPO molecules (Table S3) from the EPR line broadening caused by magnetic dipole-dipole interactions of unpaired electrons (ΔH_d). There are two methods usually used for such calculations.

According to Sackmann [7], the mean distance between paramagnetic centers of radicals is given by equation 1:

$$\mathrm{x}\,[\text{Å}] = \sqrt[3]{30000/\Delta H_d\,[G]} \tag{1}$$

We applied this equation in the same manner as Ottaviani et al. [8] for nitroxide radicals adsorbed on zeolites (the restricted motion case). The ΔH_d value was approximated using ΔH_{pp} and ΔH^0_{pp}, which are peak-to-peak linewidths with the dipolar broadening present and absent, respectively, of the central line of the nitrogen-14 hyperfine triplet. Thus, the final equation was the following:

$$\mathrm{x}\,[nm] = 0.1 \times \sqrt[3]{30000/\left(\Delta H_{pp} - \Delta H^0_{pp}\right)[G]} \tag{2}$$

where ΔH_{pp} was the actual linewidth and ΔH^0_{pp} was the linewidth measured for 0.5 mM solution of TEMPO in the appropriate solvent. For CH and BuCl the ΔH^0_{pp} values were equal to 3.2 and 3.56 G, respectively. The mean distances between TEMPO molecules were calculated only for a few initial radical concentrations, where the Heisenberg exchange rate did not exceed, or exceeded only slightly, the value of 5×10^7 s^{-1}, taken by us as the limit of slow spin exchange [9].

The obtained values were then compared with the mean distances between TEMPO molecules calculated with another method, introduced by Kokorin et al. [10]. It is based on the experimental parameter d_1/d, which can only be calculated from a rigid-type (slow-motion) spectrum with clearly visible hyperfine structure. The d_1 and d values are defined as appropriate distances on the intensity scale of the derivative EPR signal: d_1 is measured between the extreme points of the lateral lines of the nitrogen-14 hyperfine triplet, while d is a peak-to-peak intensity of its central line. The relationship between the d_1/d parameter and the mean distance x between unpaired electrons of adjacent TEMPO molecules was introduced by Ionita et al. in the form of the following empirical equation [11]:

$$d_1/d = a_1 {*} e^{-a_2(x-a_3)} + a_4 + a_5/x \tag{3}$$

where the parameters a_1-a_5 equal 0.8050, 3.0150 nm^{-1}, 0.8736 nm, 0.5145 and 0.06824 nm, respectively. Again, the interspin distances were calculated only in the cases, for which the spin exchange rate was below the aforementioned threshold value.

The calculations were not possible for component 1 of HA83/TEMPO, because already for the lowest TEMPO concentration in the CH and BuCl adsorption solutions, the SE rate was 3.0×10^8 s^{-1} and 1.6×10^8 s^{-1}, respectively, which is far over the acceptable value of 5.0×10^7 s^{-1}, limiting the slow exchange case [9]. Therefore, component 1 in all the EPR spectra of HA83/TEMPO appeared with poorly resolved or unresolved HFS. However, such fast SE implies that the TEMPO molecules adsorbed on HA83 are close to each other, even at a low radical loading. On the other hand, the TEMPO molecules adsorbed on HA83 but responsible for component 2, did not qualify for the restricted motion case, so the calculation of distances between their paramagnetic centers was inadequate.

As concerns HA259/TEMPO (only component 1), the calculated interspin distances do not cover the whole TEMPO concentration range (Table S4), because only selected cases fulfilled the restricted-motion condition and the slow SE requirement. For BuCl, method I gives decreasing values from 1.64 nm to 1.28 nm in the 0.5-8 mM concentration range, while method II gives decreasing values from 1.39 nm to 1.03 nm in the 0.5-6 mM concentration range. This decrease of interspin distances with increasing TEMPO loading was expected, but such short interspin distances for low TEMPO loadings look unusual. For CH, the mean distance between the adsorbed TEMPO molecules is also small. For the 0.5 and 1 mM solutions we got 1.24 and 1.34 nm using method I, and 1.03 and 1.09 nm using method II, respectively.

Table S4. Mean distances [nm] between paramagnetic centers of the TEMPO molecules adsorbed on HA259 (component 1), calculated using the ΔH_{pp} linewidth of the TEMPO triplet central line (method I) and the d_1/d parameter (method II). The calculations were done for the spectra with the resolved hyperfine structure corresponding to the restricted motion case. Another limitation was that the Heisenberg spin exchange rate did not exceed, or exceeded only slightly, the cutoff value of 5×10^7 s^{-1} for the slow exchange case.

Initial TEMPO concentration [mM]	1-chlorobutane		cyclohexane	
	Method I	Method II	Method I	Method II
**0.5**	1.64	1.39	1.24	1.03
**1**	1.61	1.27	1.34	1.09
**2**	1.50	1.15	- [a]	- [a]
**3**	1.48	1.08	- [a]	- [a]
**4**	1.44	1.06	- [a]	- [a]
**5**	1.35	1.03	- [a]	- [a]
**6**	1.34	1.03	- [a]	- [a]
**7**	1.29	-	- [a]	- [a]
**8**	1.28	-	- [a]	- [a]

[a] Unresolved hyperfine structure, a broad single line.

References

1. Altenbach – Multicomponent. Available online: https://sites.google.com/site/altenbach/labview-programs/epr-programs/multicomponent (accessed on 14 December 2021).
2. Katter, U.J.; Hill, T.; Risse, T.; Schlienz, H.; Beckendorf, M.; Kluner, T.; Hamann, H.; Freund, H.J. Adsorption of the stable radical di-tert-butyl nitroxide (DTBN) on an epitaxially grown Al_2O_3 film. *J. Phys. Chem. B* **1997**, *101*, 552–560. 10.1021/jp963104y
3. Grampp, G.; Landgraf, S.; Rasmussen, K.; Strauss, S. Dimerization of organic free radicals in solution. 1. Temperature dependent measurements. *Spectrochim. Acta A Mol. Biomol. Spectrosc.* **2002**, *58*, 1219–1226. 10.1016/s1386-1425(01)00712-0
4. Vegas, A. Cations in inorganic solids. *Crystallogr. Rev.* **2000**, *7*, 189–283. 10.1080/08893110008044245
5. Golubev, V.A.; Sen, V.D.; Kulyk, I.V.; Aleksandrov, A.L. Mechanism of the oxygen disproportionation of di-tert-alkylnitroxyl radicals, *Izv. Akad. Nauk SSSR, Ser. Khim.* **1975**, *10*, 2235–2243.
6. Yurpalov, V.L.; Fedorova, E.D.; Drozdov, V.A.; Lavrenov, A.V. Evaluation of the acidic properties of the B_2O_3–Al_2O_3 and Pt/B_2O_3–Al_2O_3 systems by spin probe EPR spectroscopy and their correlation with the occurrence of the joint hydroisomerization of heptane and benzene. *Kinet. Catal.* **2016**; *57*, 540–545.
7. Sackmann, E.; Träuble, H. Studies of the crystalline-liquid crystalline phase transition of lipid model membranes. II. Analysis of electron spin resonance spectra of steroid labels incorporated into lipid membranes. *J. Am. Chem. Soc.* **1972**, *94*, 4492–4498. 10.1021/ja00768a014

8. Ottaviani, M.F.; Garcia-Garibay, M.; Turro, N.J. TEMPO radicals as EPR probes to monitor the adsorption of different species into X zeolite. *Colloids Surf. A* **1993**, *72*, 321–332. 10.1016/0927-7757(93)80482-T
9. Sachse, J.H.; Marsh D. Notes: Line intensities in spin-exchanged nitroxide ESR spectra. *J. Magn. Reson.* **1986**, *68*, 540–543. 10.1016/0022-2364(86)90343-4
10. Kokorin, A.I. Forty years of the d1/d parameter. In *Nitroxides – theory, experiments and applications*, 1st ed.; Kokorin, A.I., Eds.; InTech Open: Rijeka, Croatia, 2012.; pp. 113–164.
11. Ionita, P.; Caragheorgheopol, A.; Gilbert, B.C.; Chechik, V. Dipole−dipole interactions in spin-labeled Au nanoparticles as a measure of interspin distances. *J. Phys. Chem. B* **2005**, *109*, 3734–3742. 10.1021/jp045696n
